# Supplementary material for: Genome-wide association identifies seven loci for pelvic organ prolapse in Iceland and the UK Biobank
Source: Commun Biol. 2020 Mar 17;3:129. doi: 10.1038/s42003-020-0857-9 (PMC7078216; doi:10.1038/s42003-020-0857-9)
Supplement: Supplementary file 1 — Description of Supplementary Data [file 42003_2020_857_MOESM1_ESM.pdf]

Description of Supplementary Data

Genome-wide association identifies seven loci for pelvic organ prolapse in Iceland and the UK Biobank

Olafsdottir et al.

Supplementary Data 1: Demographic information for POP cases and controls in Iceland and the UKBiobank

Supplementary Data 2: *P*-value thresholds for genome-wide significance for variants in different annotations classes

Supplementary Data 3: Association results for variants reaching genome-wide significance in meta-analysis of pelvic organ prolapse

Supplementary Data 4: Association results for the lead variant (rs3791675) at the *EFEMP1* locus on chromosome 2 and a secondary signal detected with a conditional analysis in a meta-analysis of pelvic organ prolapse

Supplementary Data 5: Sequence variants reported in previous GWAS studies on POP tested for association with POP in the data from Iceland and UK biobank

Supplementary Data 6: Association of POP with variants previously reported to associate with POP in meta-analyses of candidate-gene studies

Supplementary Data 7: A summary of surgical procedures and the corresponding codes from the classification of surgical procedures available in the UKB and Icelandic data

Supplementary Data 8: Association between POP variants and POP phenotype by definition of POP cases in UK, Iceland and in the combined analysis

Supplementary Data 9: Shown are associations of POP index variants with traits previously reported for associations at each of the eight loci (*) and traits detected using combined data from Iceland and UKB

Supplementary Data 10: Previously reported variants that are in LD (r^2>0.5) with the POP variants detected in the current study and the associated traits

Supplementary Data 11: Association of POP index variants with POP phenotypes where leiomyoma and endometriosis cases have been excluded

Supplementary Data 12: Co-localization analysis

Supplementary Data 13: Association of POP with 32 reported leiomyoma variants

Supplementary Data 14: Association of POP with five reported gestational duration variants

Supplementary Data 15: Association of POP with 27 reported endometriosis variants

Supplementary Data 16: Association of POP with 14 reported sequence variants for Barrett's oesophagus and esophageal adenocarcinoma (BE and AE combined)

Supplementary Data 17: Association of POP with 145 reported prostate cancer variants

Supplementary Data 18: Association of POP with four reported inguinal hernia variants

Supplementary Data 19: Association of POP with 3,273 reported height variants

Supplementary Data 20: Association of POP with 52 reported lung function variants

Supplementary Data 21: Association of POP with 70 reported waist circumference (BMI-adjusted) variants

Supplementary Data 22: Association of POP with 23 reported benign prostatic hyperplasia variants

Supplementary Data 23: Association of POP with 152 reported BMI variants

Supplementary Data 24: Association of POP with three reported fertility variants

Supplementary Data 25: Association results for POP variants using data from UK Biobank with BMI and number of children added separately as covariates

Supplementary Data 26: Associations of polygenic risk scores (PRS) for chip-genotyped cases and controls in Iceland and UKB with POP and traits previously reported as risk factors for POP; body mass index (BMI) and number of children

Supplementary Data 27: Association results for POP variants in or nearby *EFEMP1* and aging-associated phenotypes

Supplementary Data 28: The proportion of diagnosed cases with POP within each of the 9 subgroups of ICD-10 N81 in the data from UK Biobank and from Iceland

Supplementary Data 29: Shown are associations of POP index variants with traits previously reported for associations at each of the eight loci (*) and traits detected using data from Iceland, UKB and the combined datasets
