# Supplementary material for: Genome-wide association identifies seven loci for pelvic organ prolapse in Iceland and the UK Biobank
Source: Commun Biol. 2020 Mar 17;3:129. doi: 10.1038/s42003-020-0857-9 (PMC7078216; doi:10.1038/s42003-020-0857-9)
Supplement: Supplementary file 2 — Supplementary Information [file 42003_2020_857_MOESM2_ESM.pdf]

# Supplementary Information

## Genome-wide association identifies seven loci for pelvic organ prolapse in Iceland and the UK Biobank

Olafsdottir et al.

# Supplementary Figures

**Supplementary Figure 1 A quantile-quantile plot (QQ-plot) of the *P*-values (chi-square statistics corrected for relatedness and stratification using correction factor estimated from LD score regression (see Methods)) for variants from the GWAS of pelvic organ prolapse in a) Iceland, b) UK Biobank and c) UK Biobank excluding the eight lead POP variants and their correlates of $r^2$>0.05 within a 2MB window**. The reason for the large difference observed in the deviation of the *P*-values from the expected null distribution in a) and b) is the difference in statistical power between the datasets from Iceland and UKB (3,409 POP cases in Iceland and 11,601 POP cases in UKB). As shown in c), once the POP variants and their correlates are excluded from the QQ plot there is little deviation of the distribution of the test statistic from the null distribution. Sequence variants with imputation information >0.8 and minor allele frequency >0.01% are plotted in the figure. The red diagonal line represents no departure of the empirical (observed) distribution from the expected distribution of the chi-square statistics.

**a)**

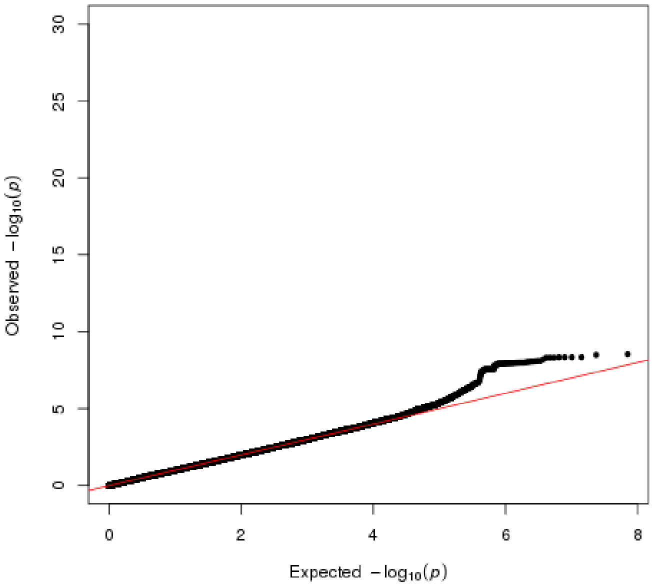

**b)**

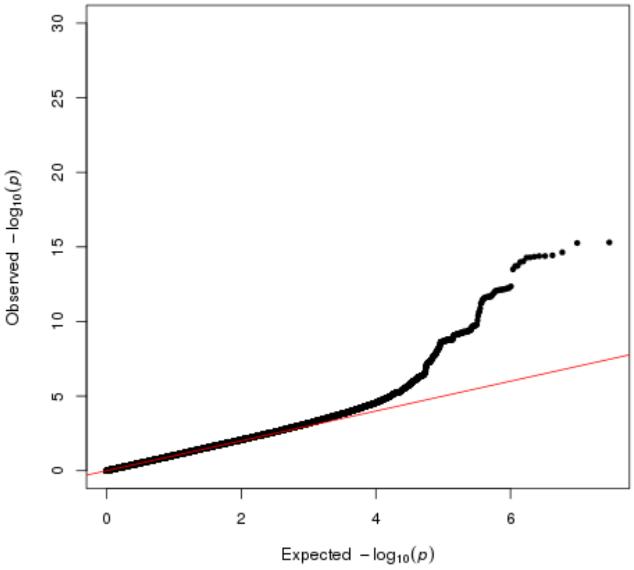

Observed $-\log_{10}(p)$
Expected $-\log_{10}(p)$
0
5
10
15
20
25
30
0
2
4
6


**c)**

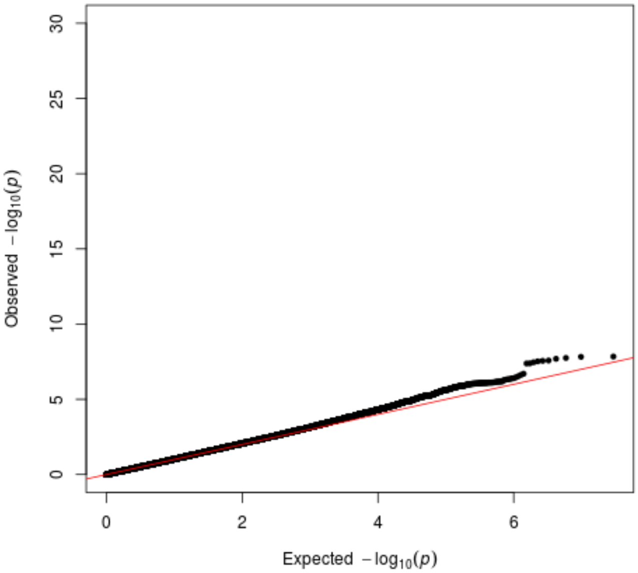

Observed $-\log_{10}(p)$
Expected $-\log_{10}(p)$
0
5
10
15
20
25
30
0
2
4
6

**Supplementary Figure 2 Regional association plots for the lead variants from the meta-analysis of pelvic organ prolapse.** *P*-values ($-\log_{10}$) of variant associations with pelvic organ prolapse (POP) in the meta-analysis are plotted against their NCBI Build 38 positions. The colors of the genomic variants reflect the linkage disequilibrium ($r^2$) with the top signal for POP in each locus in the Icelandic data set. The solid grey line indicates recombination rates from the Icelandic recombination map for males and females (Kong, A. et al, *Nature* **467,** 1099-1103 (2010)). Known genes are shown with horizontal blue lines and exons as rectangles using data from the UCSC genes track in the UCSC Genome Browser. Only high-quality markers (imputation information > 0.8) are displayed with a 500 kb window around the lead variant. Only markers found in both datasets are plotted to avoid inconsistency in calculated *P*-values by sample size. Panel d) displays the secondary signal detected with the conditional analysis that was performed.

**a)**

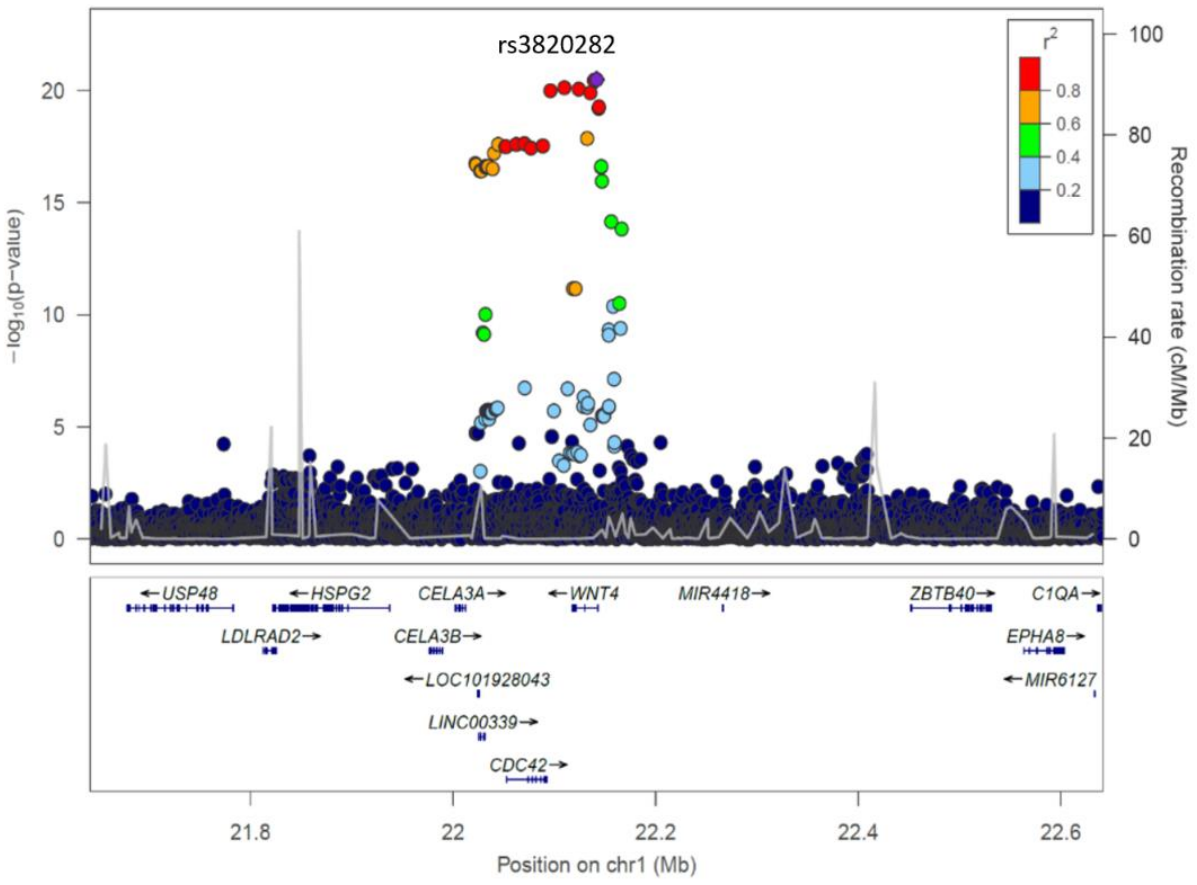

**b)**

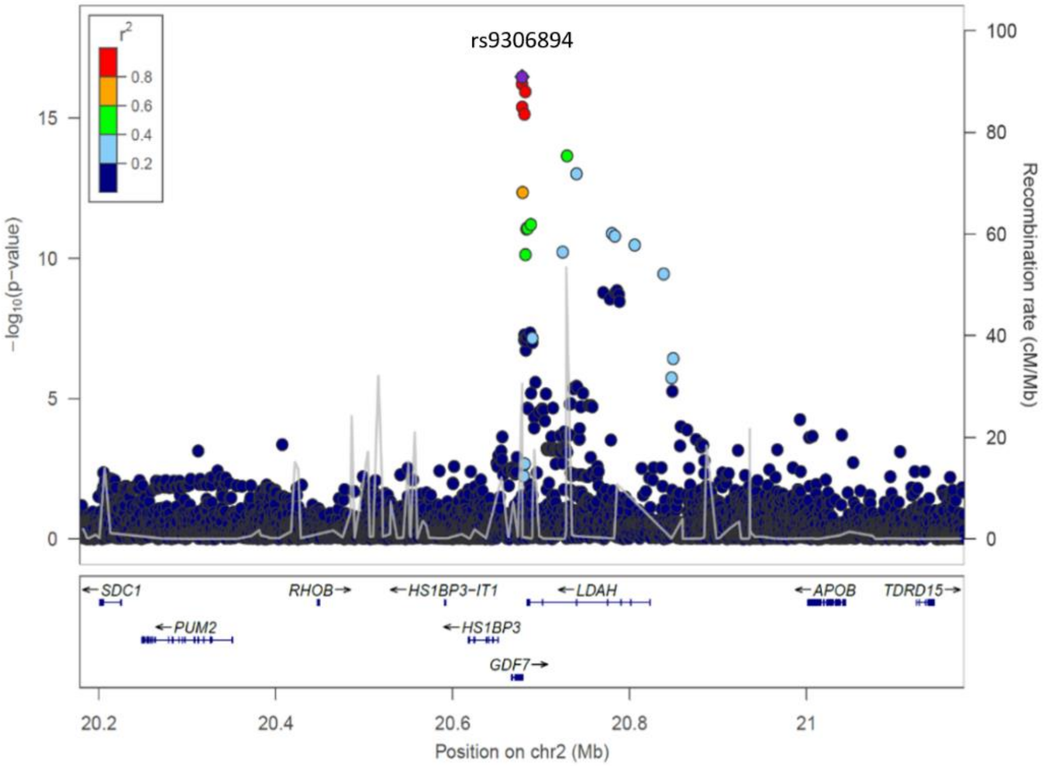
rs9306894
$r^2$
0.8
0.6
0.4
0.2
15
10
5
0
-log10(p-value)
100
80
60
40
20
0
Recombination rate (cM/Mb)
SDC1
PUM2
RHOB
HS1BP3-IT1
HS1BP3
GDF7
LDAH
APOB
TDRD15
20.2
20.4
20.6
20.8
21
Position on chr2 (Mb)

**c)**

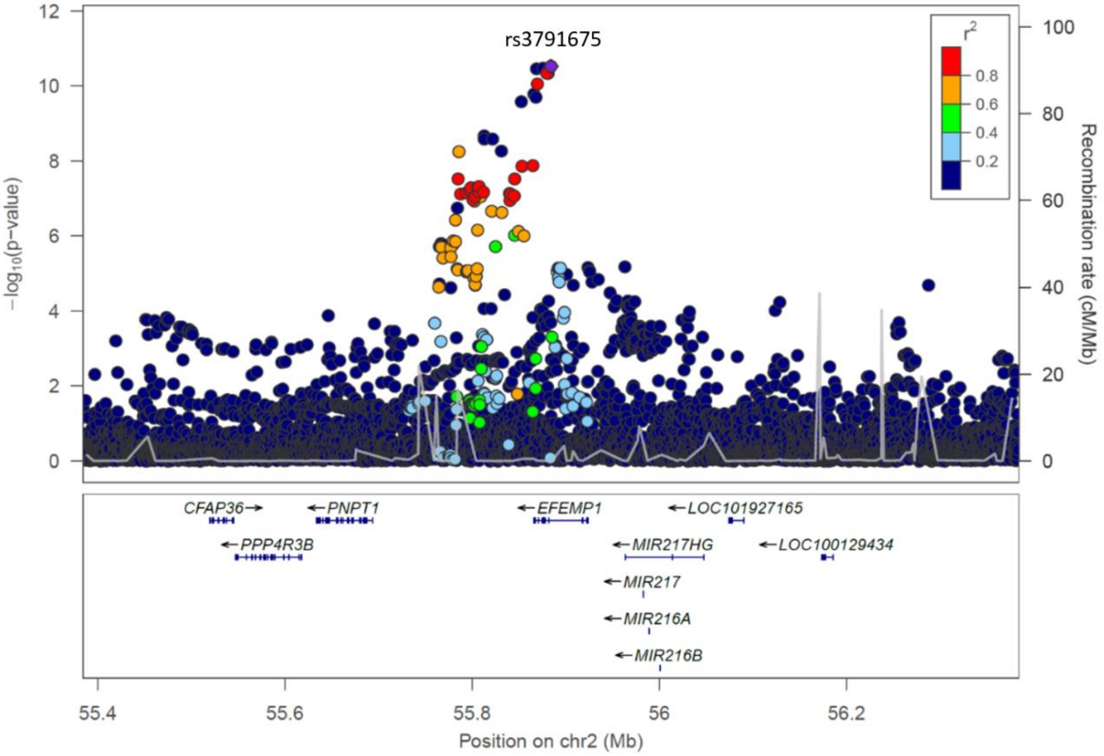
rs3791675
$r^2$
0.8
0.6
0.4
0.2
12
10
8
6
4
2
0
-log10(p-value)
100
80
60
40
20
0
Recombination rate (cM/Mb)
CFAP36
PPP4R3B
PNPT1
EFEMP1
LOC101927165
MIR217HG
LOC100129434
MIR217
MIR216A
MIR216B
55.4
55.6
55.8
56
56.2
Position on chr2 (Mb)

**d)**

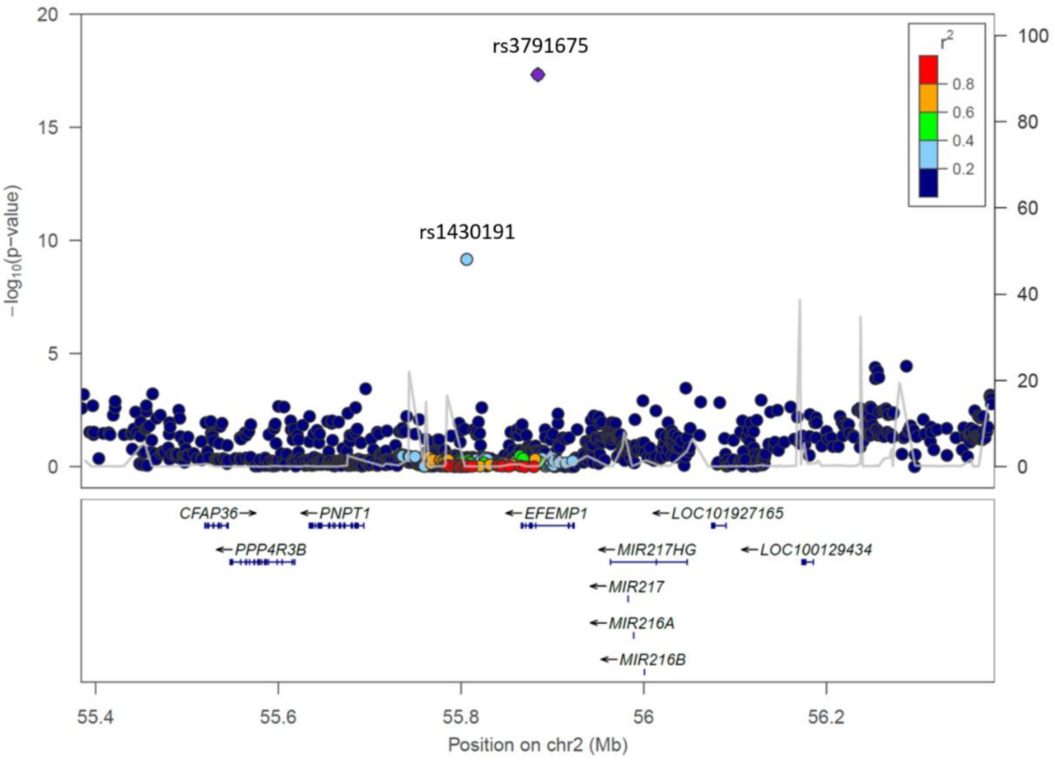
rs3791675
rs1430191
r²
0.8
0.6
0.4
0.2
−log10(p−value)
CFAP36
PNPT1
EFEMP1
LOC101927165
PPP4R3B
MIR217HG
LOC100129434
MIR217
MIR216A
MIR216B
55.4
55.6
55.8
56
56.2
Position on chr2 (Mb)

**e)**

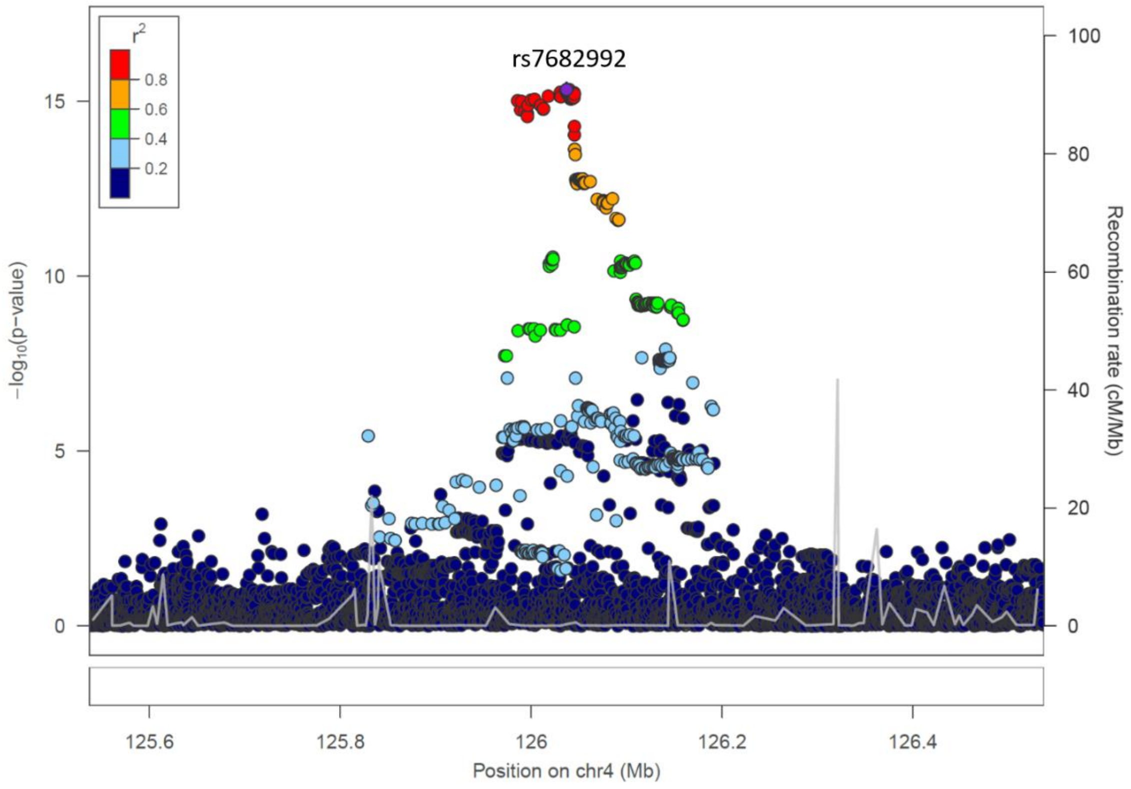
r²
0.8
0.6
0.4
0.2
rs7682992
−log10(p−value)
Recombination rate (cM/Mb)
125.6
125.8
126
126.2
126.4
Position on chr4 (Mb)

f)

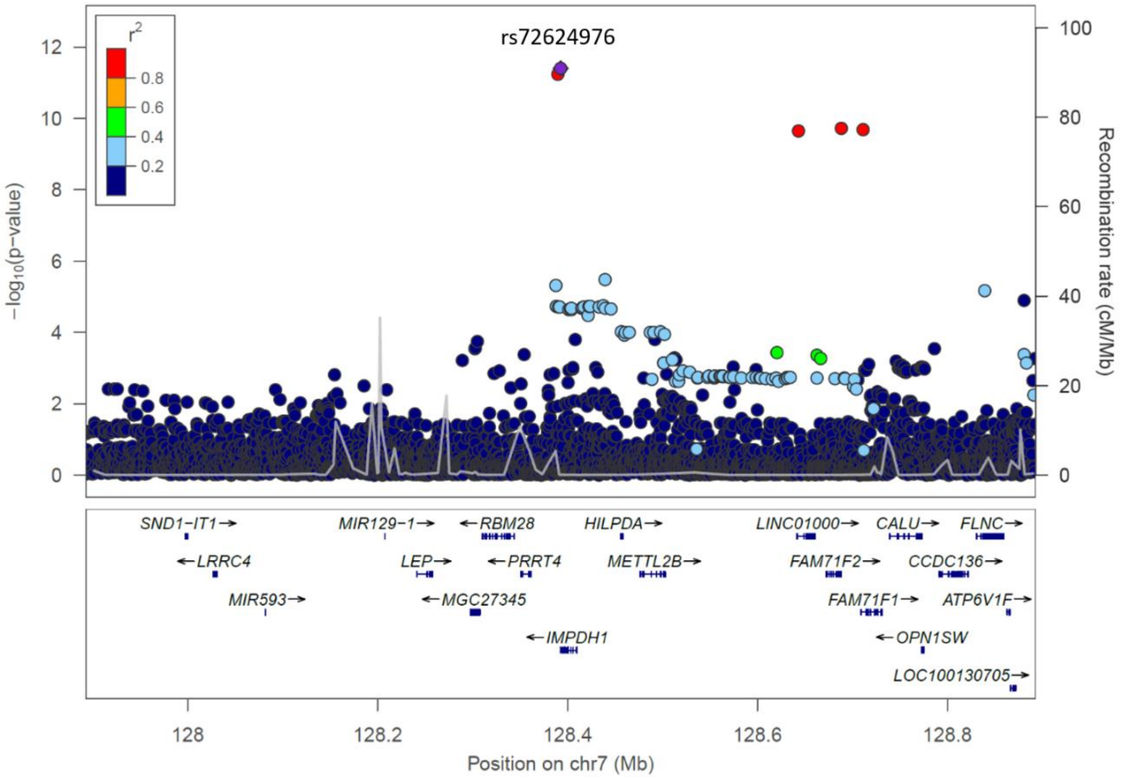
rs72624976
r2
0.8
0.6
0.4
0.2
−log10(p−value)
Recombination rate (cM/Mb)
SND1−IT1→
MIR129−1→
←RBM28
HILPDA→
LINC01000→
CALU→
FLNC→
←LRRC4
LEP→
←PRRT4
METTL2B→
FAM71F2→
CCDC136→
MIR593→
←MGC27345
FAM71F1→
ATP6V1F→
←IMPDH1
←OPN1SW
LOC100130705→
Position on chr7 (Mb)

g)

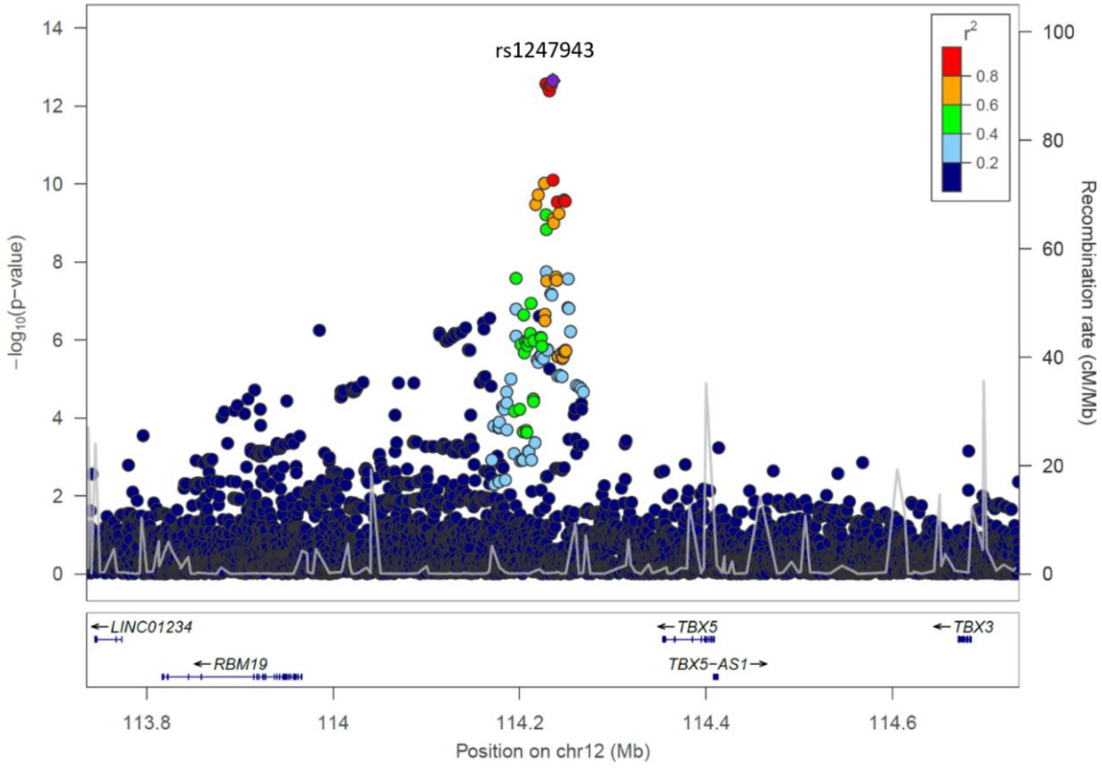
rs1247943
r2
0.8
0.6
0.4
0.2
−log10(p−value)
Recombination rate (cM/Mb)
←LINC01234
←TBX5
←TBX3
←RBM19
TBX5−AS1→
Position on chr12 (Mb)

**h)**

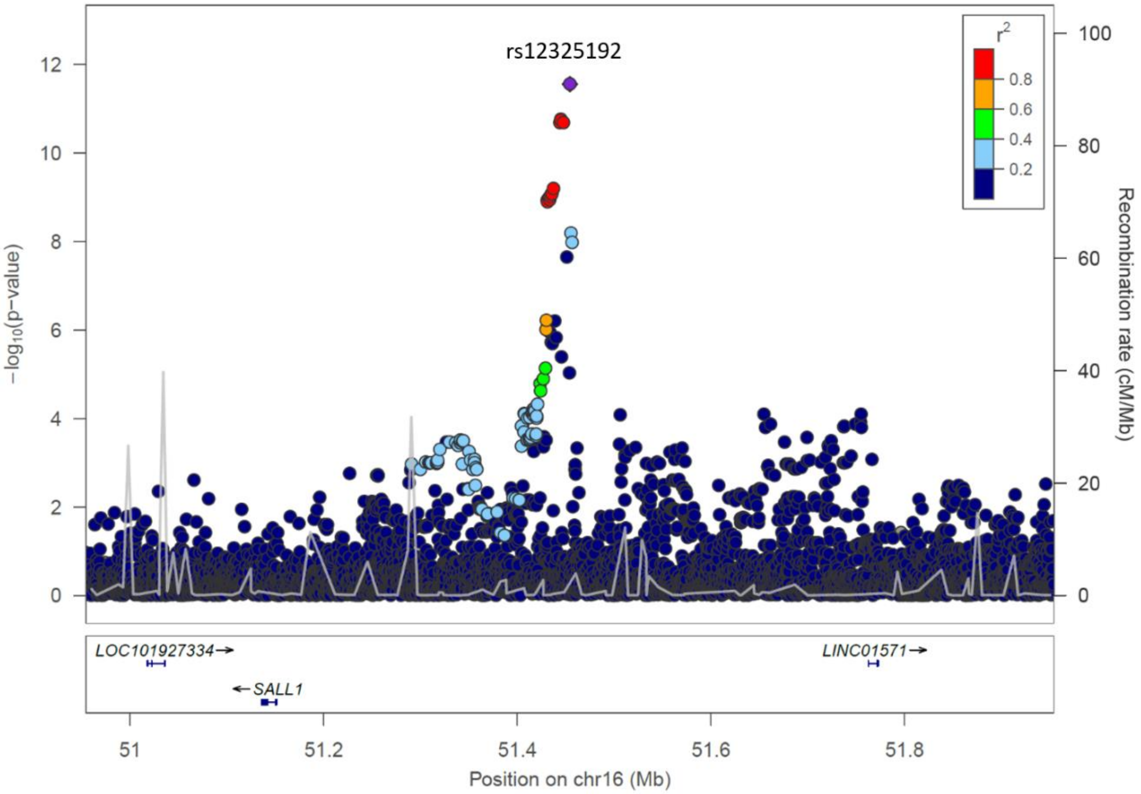
rs12325192
r²
0.8
0.6
0.4
0.2
−log10(p−value)
Recombination rate (cM/Mb)
Position on chr16 (Mb)
LOC101927334→
←SALL1
LINC01571→
51
51.2
51.4
51.6
51.8
0
2
4
6
8
10
12
20
40
60
80
100

**Supplementary Figure 3 Scatter plots for the effect estimates of a total of 3,635 published variants for nine traits against the effect estimates of those variants on POP**. On the y-axes are the natural logarithm of the odds ratios (log(OR)) from a meta-analysis of POP in Iceland and UKB and on the x-axes are the log(ORs) or betas previously reported for the nine traits. Blue dots denote variants that associate significantly with POP at the Bonferroni-corrected *P*-value < $1.4 \times 10^{-5}$ (0.05/3,591) and red dots denote variants not surpassing the threshold. Error bars denote 95% confidence intervals. Association summary results for the four reported inguinal hernia variants are reported in Supplementary Data 18. A linear fit through the data points using a simple linear regression with AF×(1-AF) as a weight is displayed where the estimated slope coefficient differs from zero at the 95% confidence level (*P*<0.05).

**a)** Shown is the logarithm of the estimated ORs of **leiomyoma** sequence variants against the logarithm of the estimated ORs on POP. Effects estimates are shown for 32 reported leiomyoma variants (Rafnar, T. et al., *Nature communications* **9**, 3636 (2018) and Välimäki, N. et al., eLife; 7:e37110 (2018)). Association summary results are also reported in Supplementary Data 13.

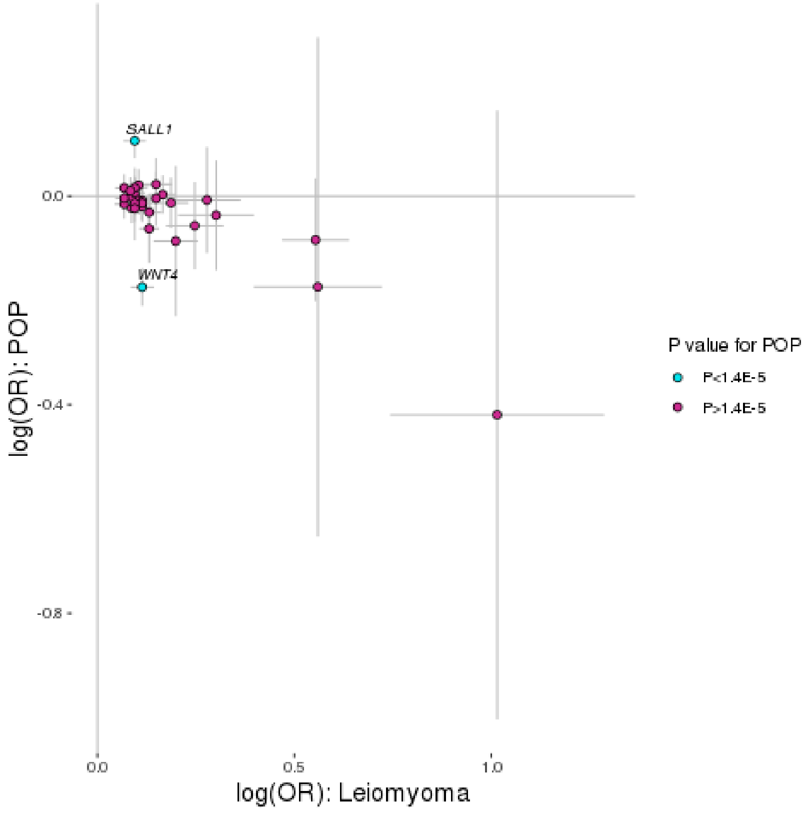

**b)** Shown are estimates of the marginal effects (betas) of **gestational duration** sequence variants against the logarithm of the estimated ORs on POP. Effects estimates are shown for five reported gestational duration variants (Zhang, G. et al. *The New England journal of medicine* **377**, 1156-1167 (2017)). Association summary results are also reported in Supplementary Data 14.

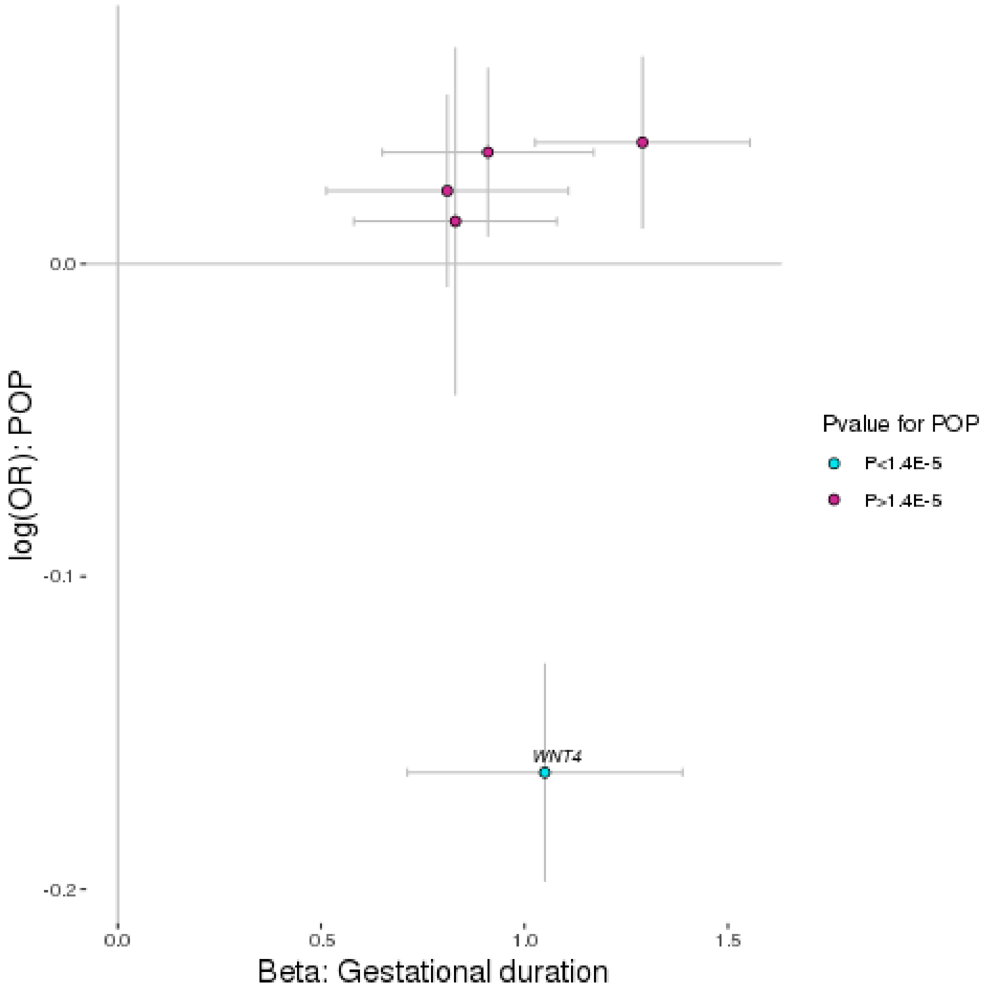

**c)** Shown is the logarithm of the estimated ORs of **endometriosis** sequence variants against the logarithm of the estimated ORs on POP. Effects estimates are shown for 27 reported endometriosis variants (Rahmioglu, N. et al., *bioRxiv*, 406967 (2018)). Association summary results are also reported in Supplementary Data 15.

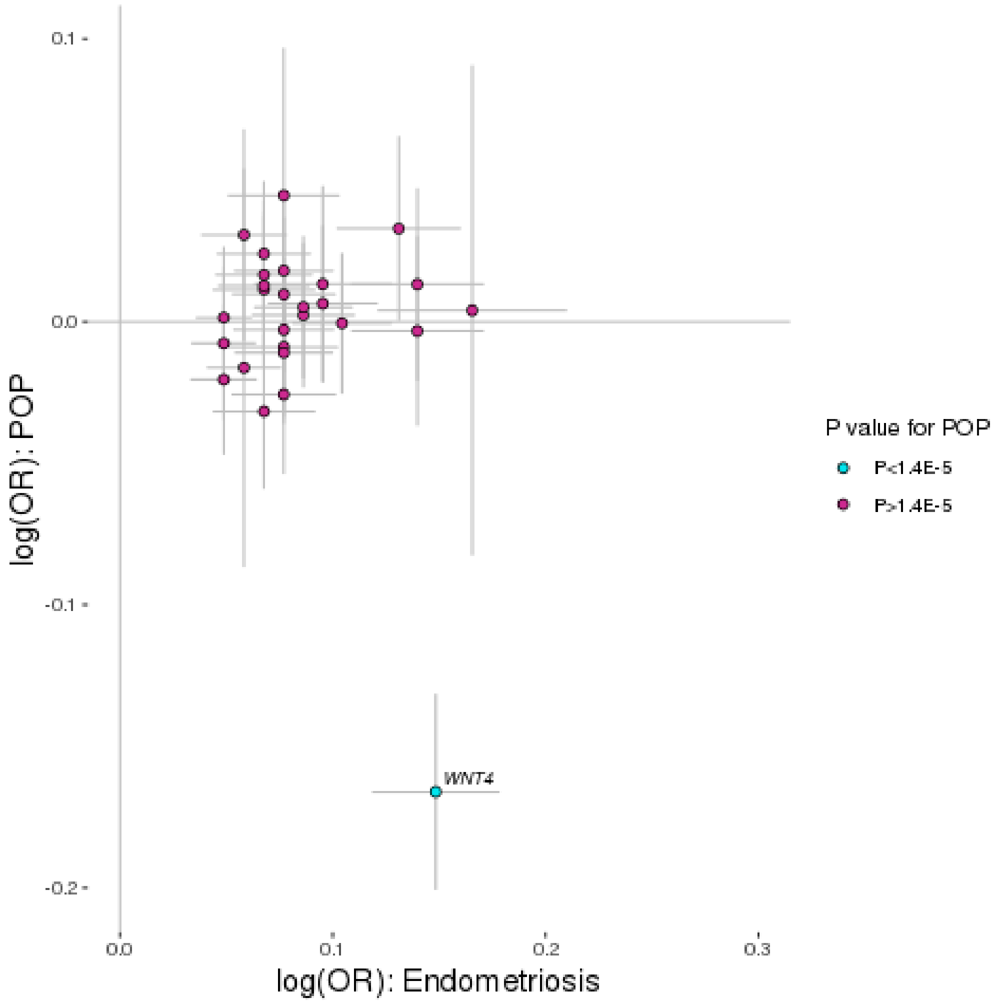

**d)** Shown is the logarithm of the estimated ORs of **prostatic cancer** sequence variants against the logarithm of the estimated ORs on POP. Effects estimates are shown for 145 reported prostatic cancer variants (Schumacher, F. R. et al., Nature genetics, **50**, 928-936 (2018)). Association summary results are also reported in Supplementary Data 17.

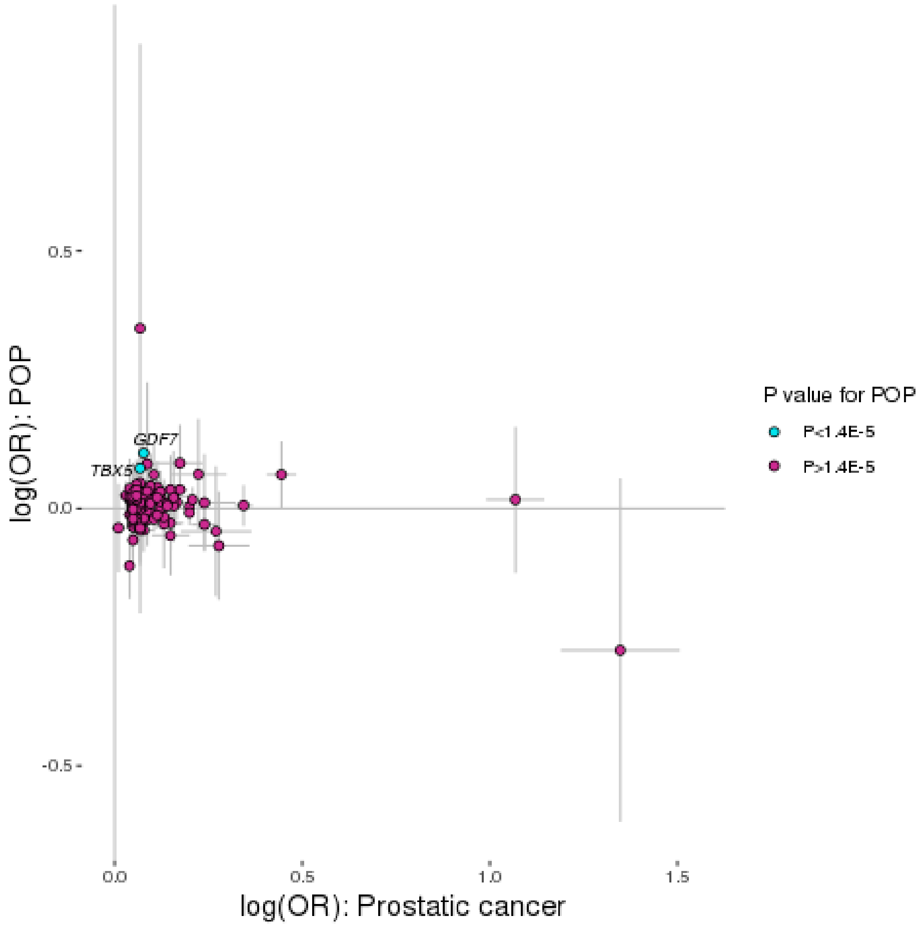

**e)** Shown is the logarithm of the estimated ORs of Barrett's oesophagus and esophageal adenocarcinoma (BE and AE combined) sequence variants against the logarithm of the estimated ORs on POP. Effects estimates are shown for 14 reported endometriosis variants (Gharahkhani, P. et al., *Lancet Oncol*., **17**, 1363-1373 (2016)). Association summary results are also reported in Supplementary Data 16.

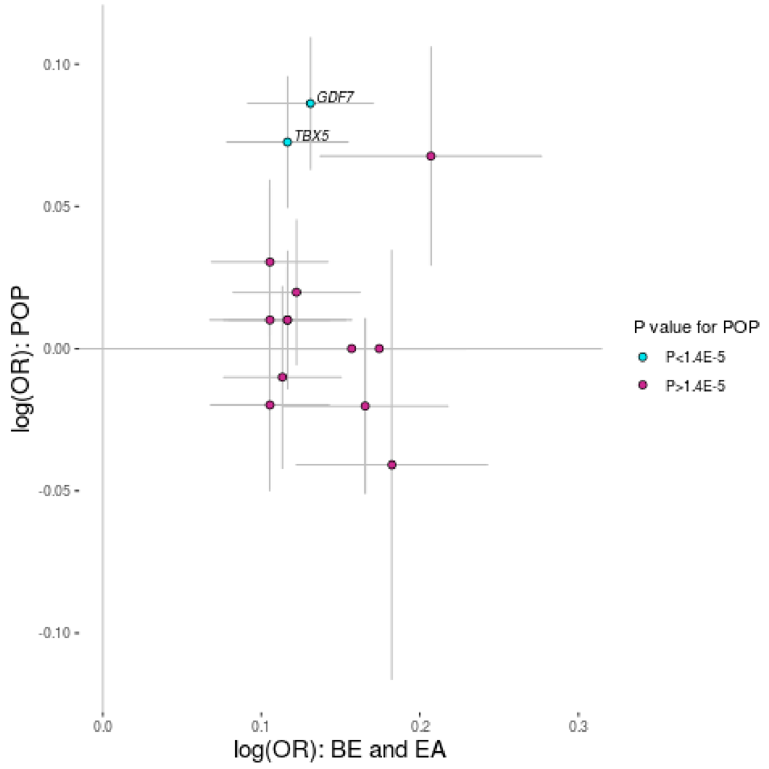

**f)** Shown are estimates of the marginal effects (betas) of **height** sequence variants against the logarithm of the estimated ORs on POP. Effects estimates are shown for 3,273 reported height variants (Yengo, L. et al., *Human molecular genetics* **27**, 3641-3649 (2018)). Association summary results are also reported in Supplementary Data 19. The slope estimate for a linear fit, using allele frequency×(1- allele frequency) as weight, is 0.038 (*P*=0.03).

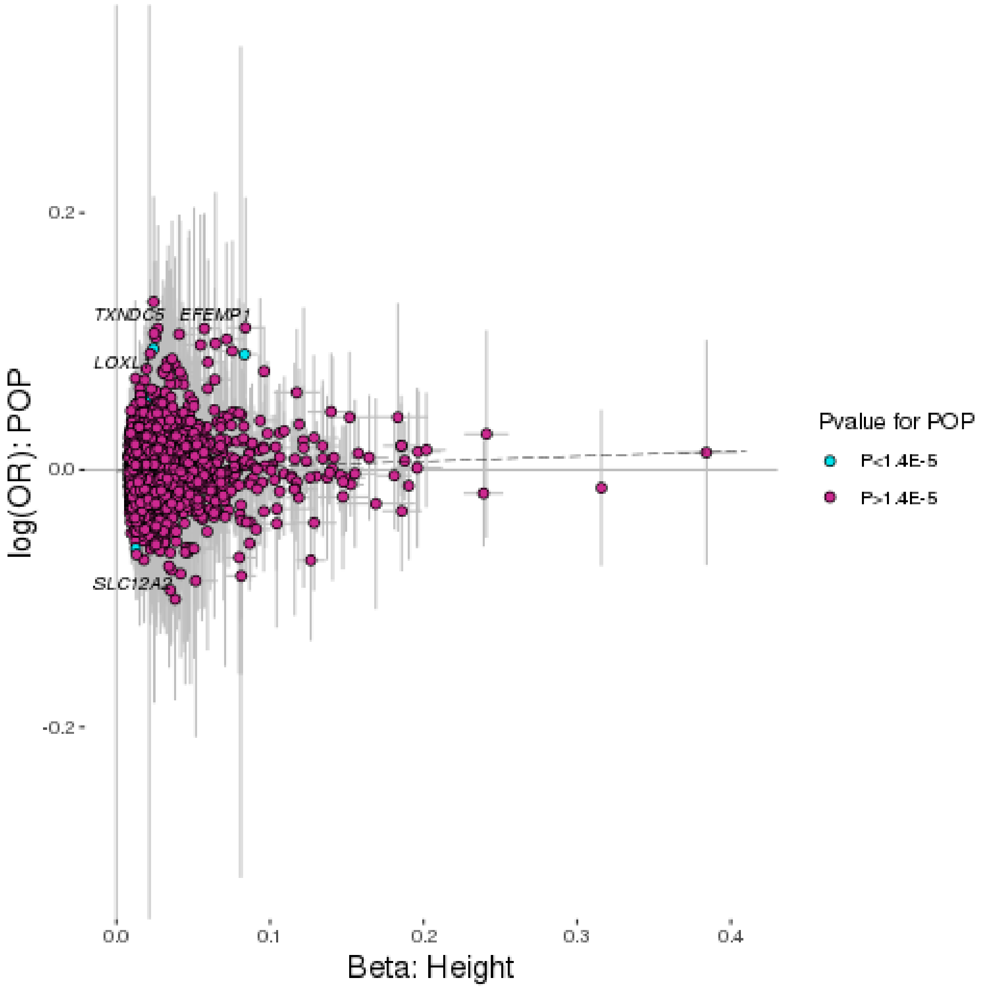

**g)** Shown are estimates of the marginal effects (betas) of **forced expiratory volume/forced vital capacity (FEV1/FVC)** sequence variants against the logarithm of the estimated ORs on POP. Effects estimates are shown for 52 reported **FEV1/FVC** variants (Wain, L. V. et al. Nature Genetics **49**, 3 (2017)). Association summary results are also reported in Supplementary Data 20.

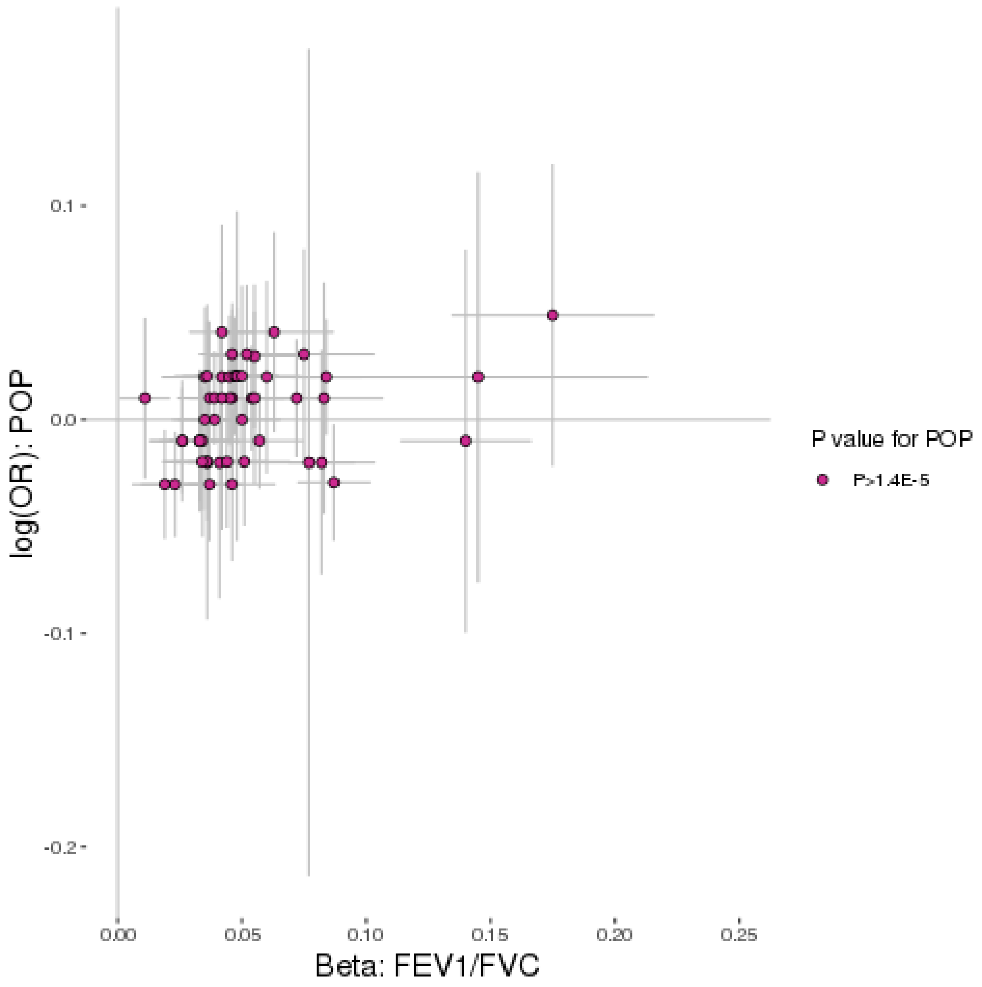

**h)** Shown are estimates of the marginal effects (betas) of **waist circumference (BMI-adjusted)** sequence variants against the logarithm of the estimated ORs on POP. Effects estimates are shown for 70 reported waist circumference variants (Shungin, D. *et al. Nature* **518**, 187-196 (2015)). The data are also shown in Supplementary Data 21.

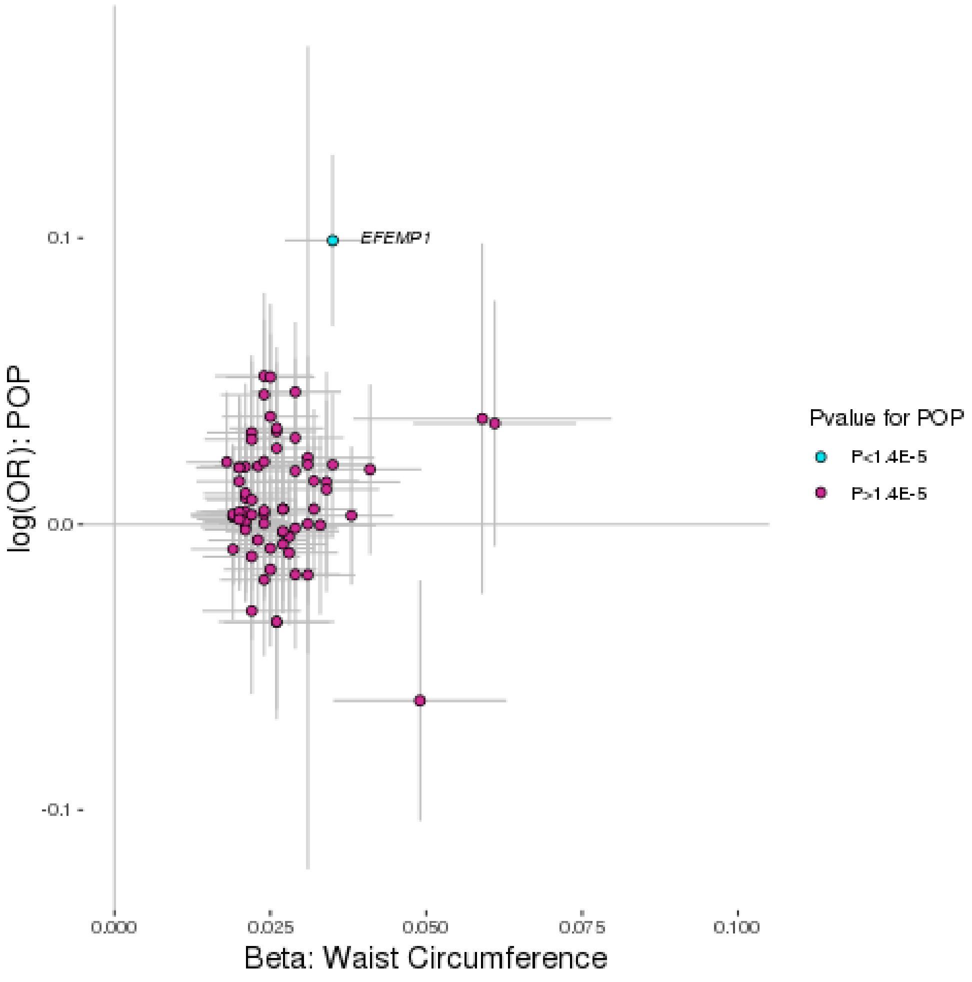

**i)** Shown is the logarithm of the estimated ORs of **benign prostatic hyperplasia (BPH)** sequence variants against the logarithm of the estimated ORs on POP. Effects estimates are shown for 23 reported BPH variants (Gudmundsson, J. et al., *Nature communications* **9,** 4568 (2018)). Association summary results are also shown in Supplementary Data 22.

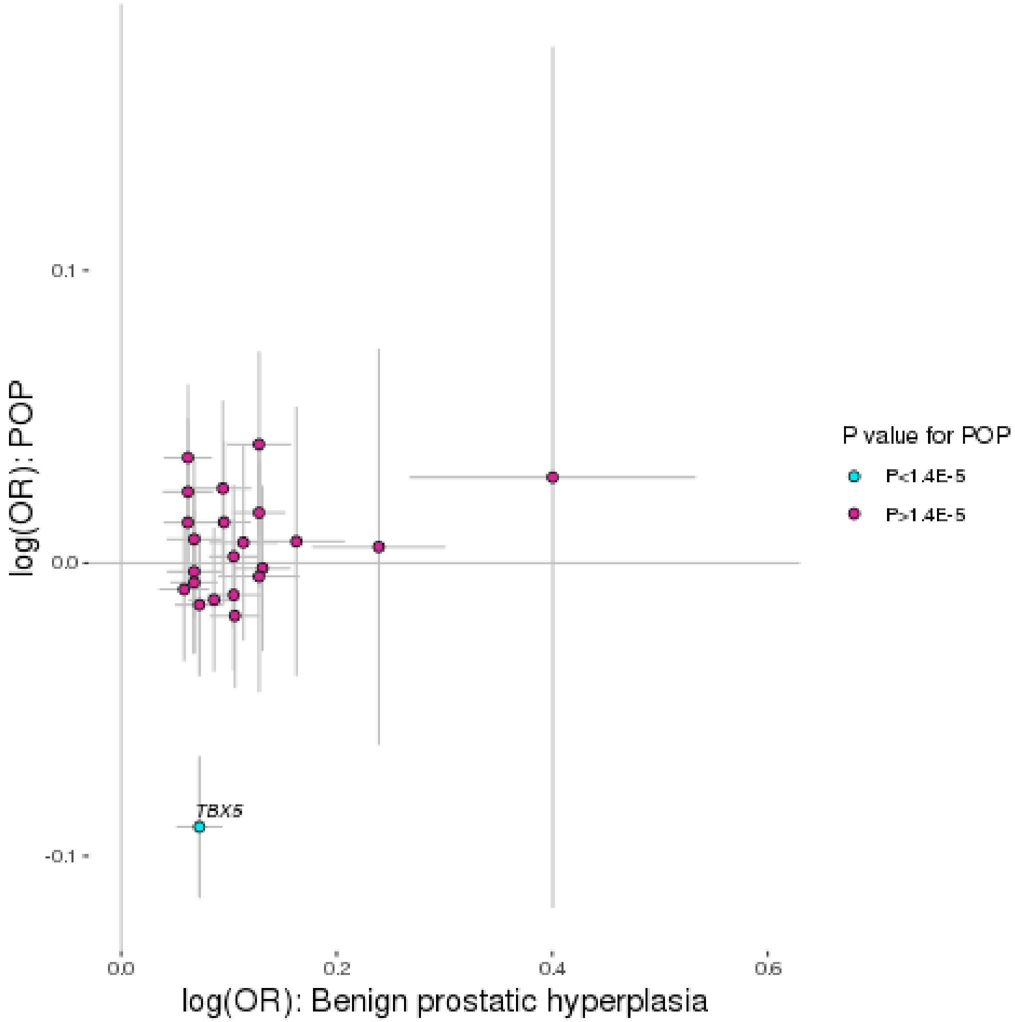

**Supplementary Figure 4 Comparison of the effect estimates of POP variants on POP against the effect estimates of those variants on the 13 traits listed in Table 2**. Effect estimates are shown for the eight index variants listed in Table 1. The traits are a) leiomyoma, b) gestational duration, c) endometriosis, d) prostate cancer, e) BE and EA, f) inguinal hernia, g) height, h) FEV1/FVC, i) carpal tunnel syndrome, j) ventral hernia, k) waist circumference, l) stress incontinence, m) benign prostatic hyperplasia. On the y-axes are the natural logarithm of the odds ratios (log(OR)) or betas from a meta-analysis of the 13 traits in Iceland and UKB (gestational duration and height in Iceland only) and on the x-axes are the log(ORs) from the meta-analysis of POP in Iceland and UKB. Blue dots denote variants that associate significantly with the traits at the Bonferroni-corrected *P*-value $< 6.3\times10^{-5}$ (0.05/800) (see Table 2) and red dots denote variants not surpassing the threshold. Error bars denote 95% confidence intervals.

**a)** Leiomyoma

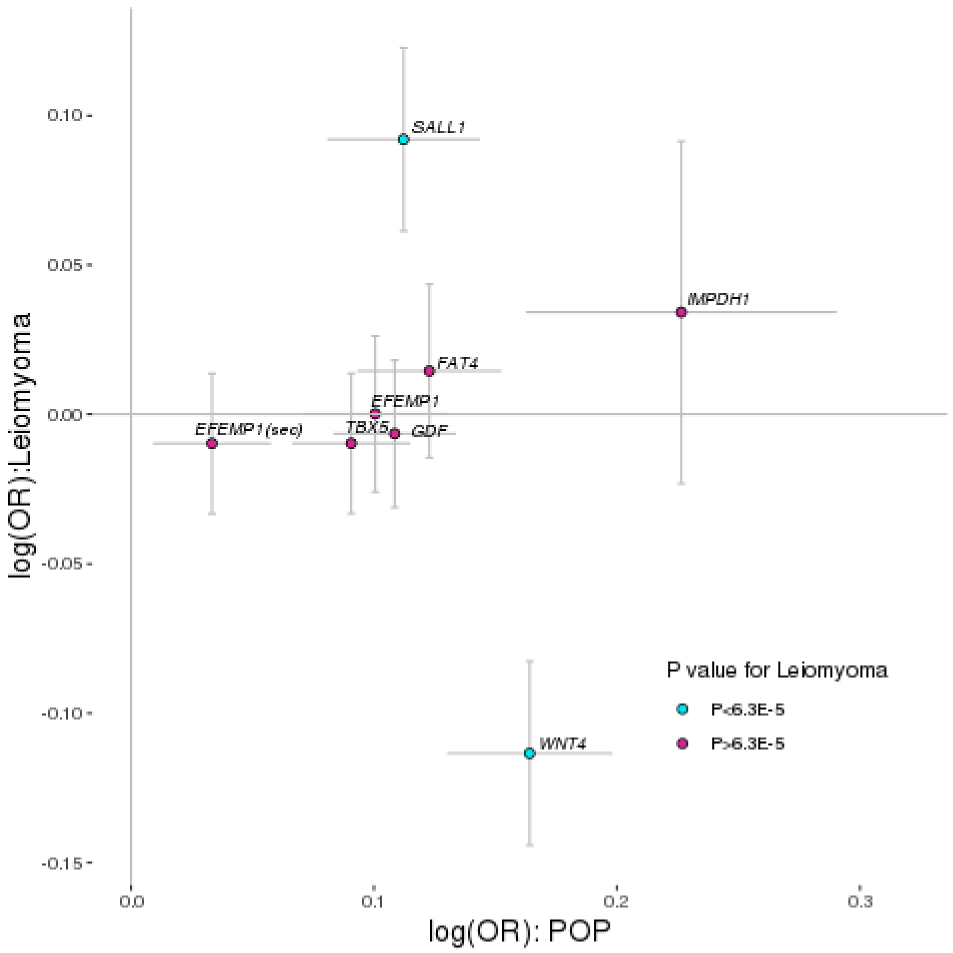

**b)** Gestational duration

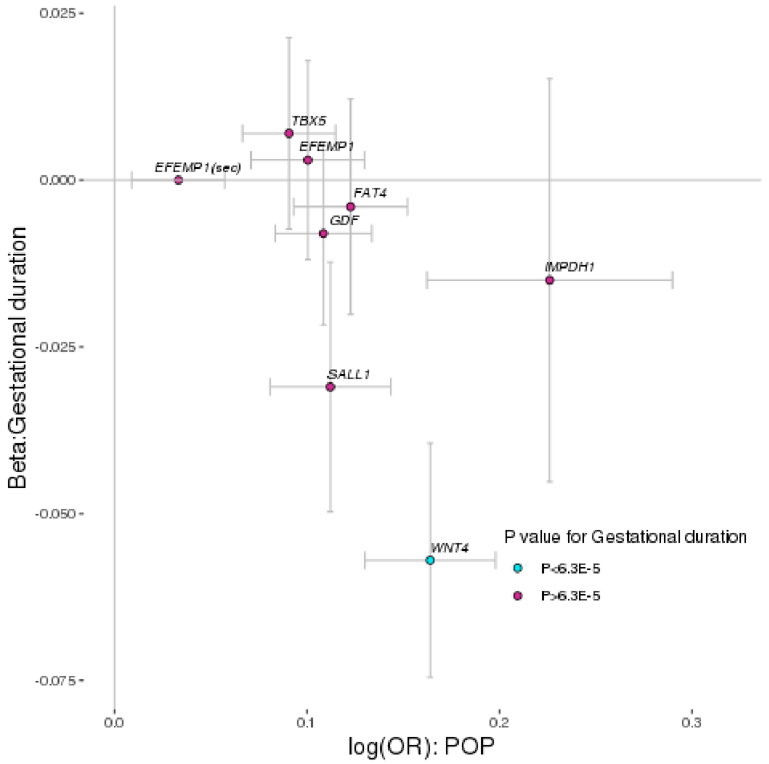


**c)** Endometriosis

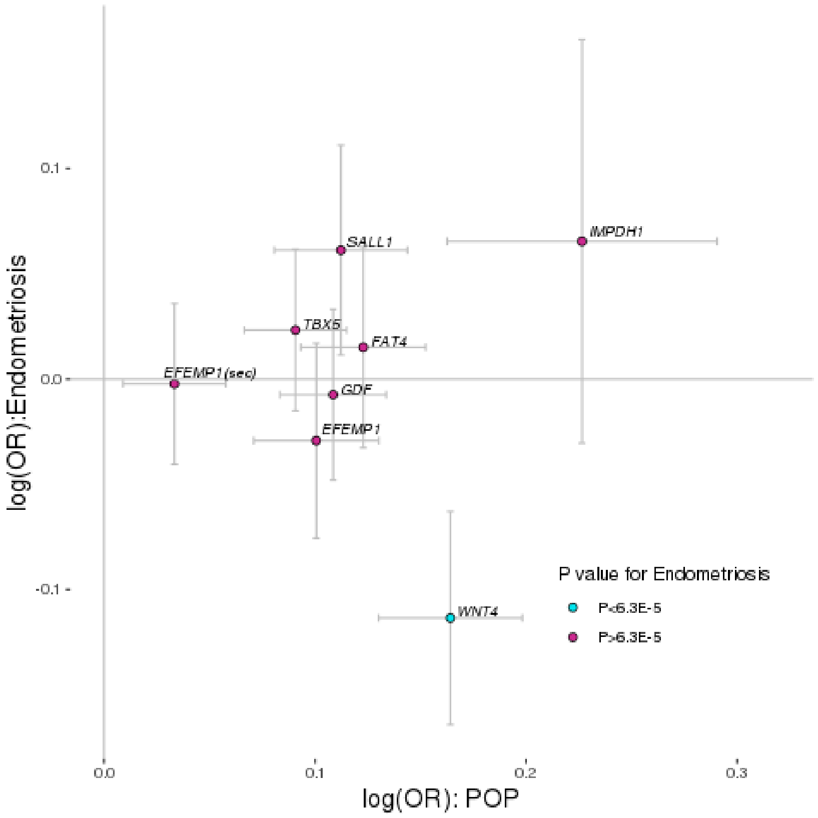

**d)** Prostate cancer

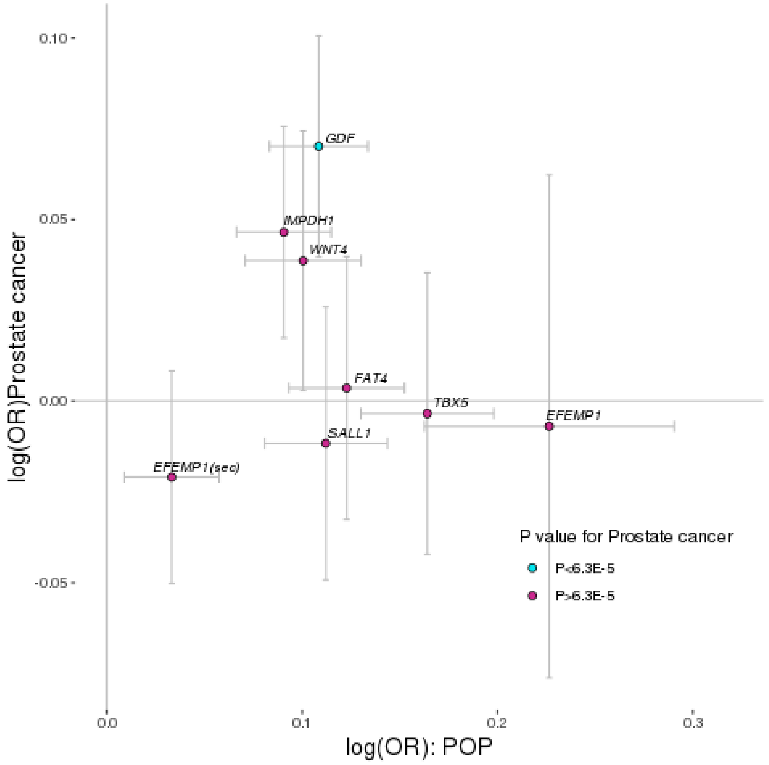


**e)** Barrett's oesophagus and esophageal adenocarcinoma

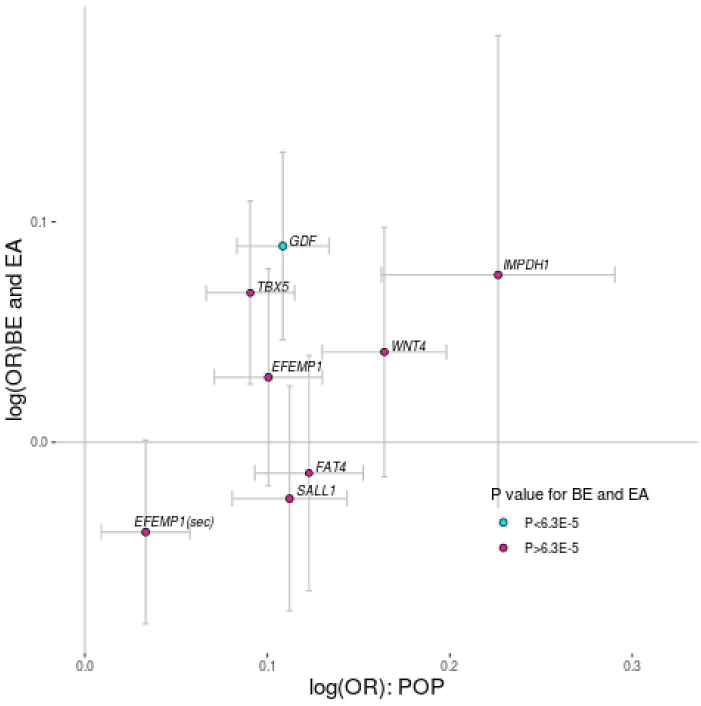

**f)** Inguinal hernia

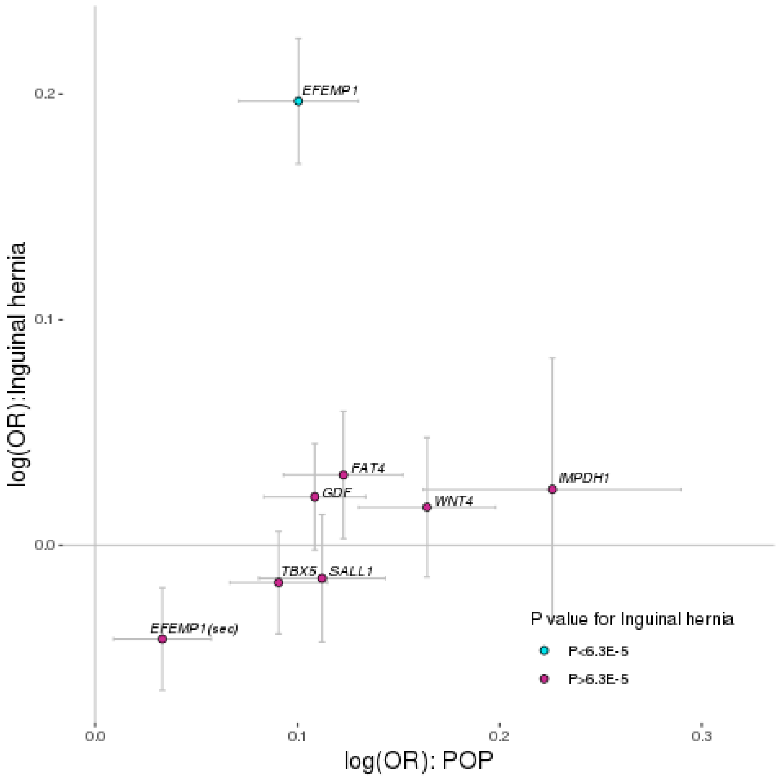


**g)** Height

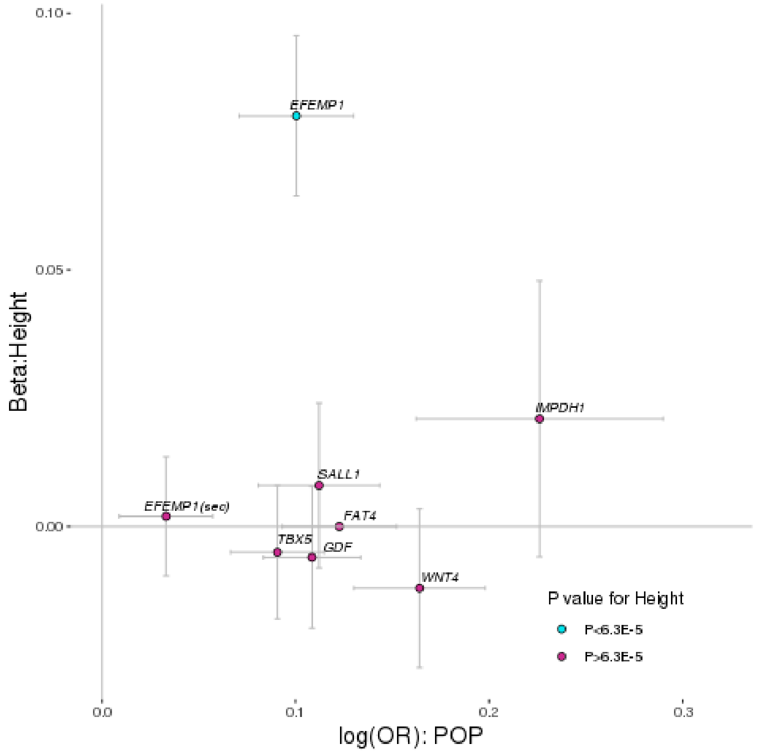

**h)** FEV1/FVC

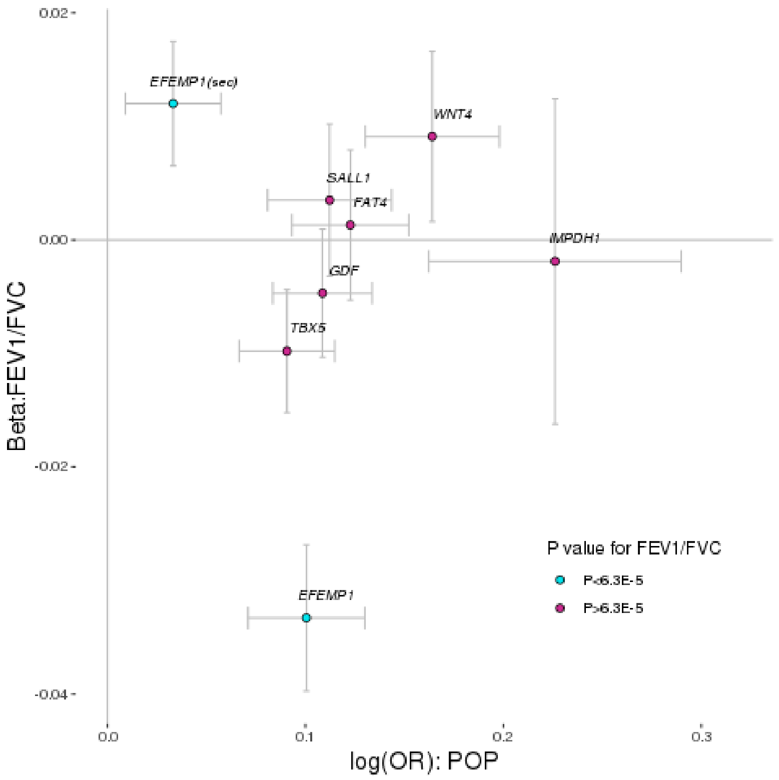


**i)** Carpal tunnel syndrome

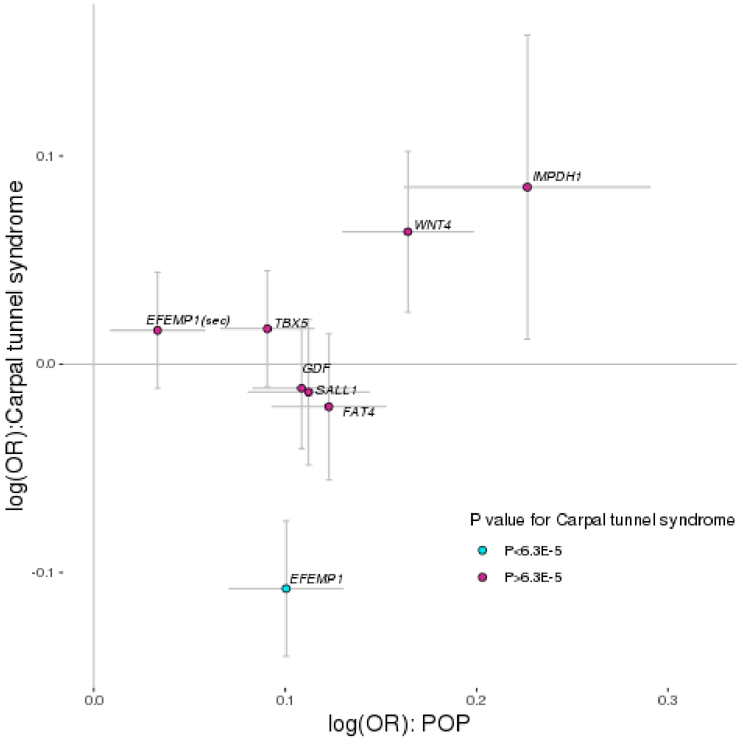

**j)** Ventral hernia

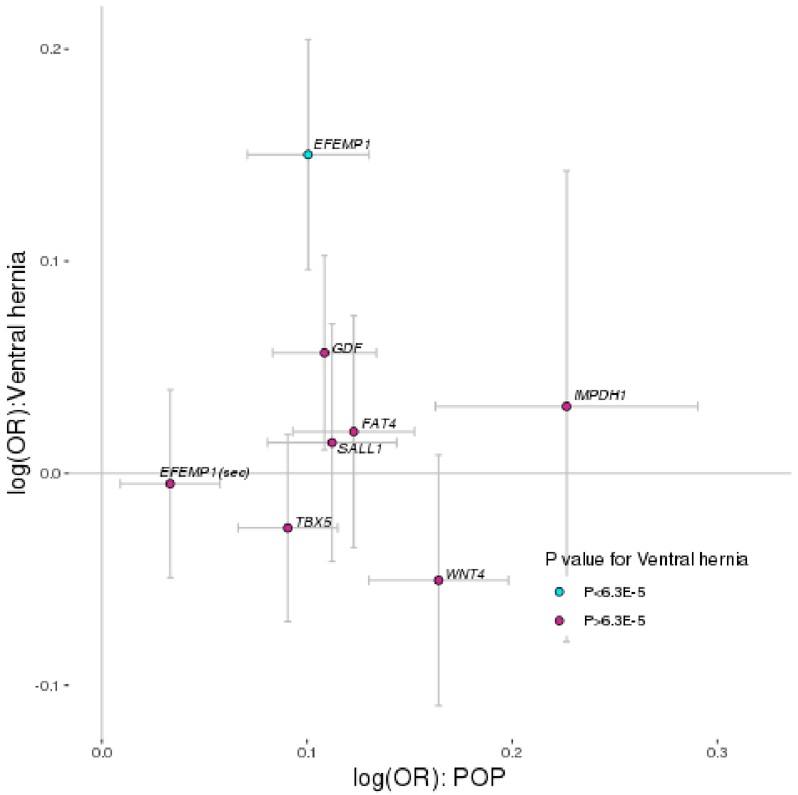


**k)** Waist circumference

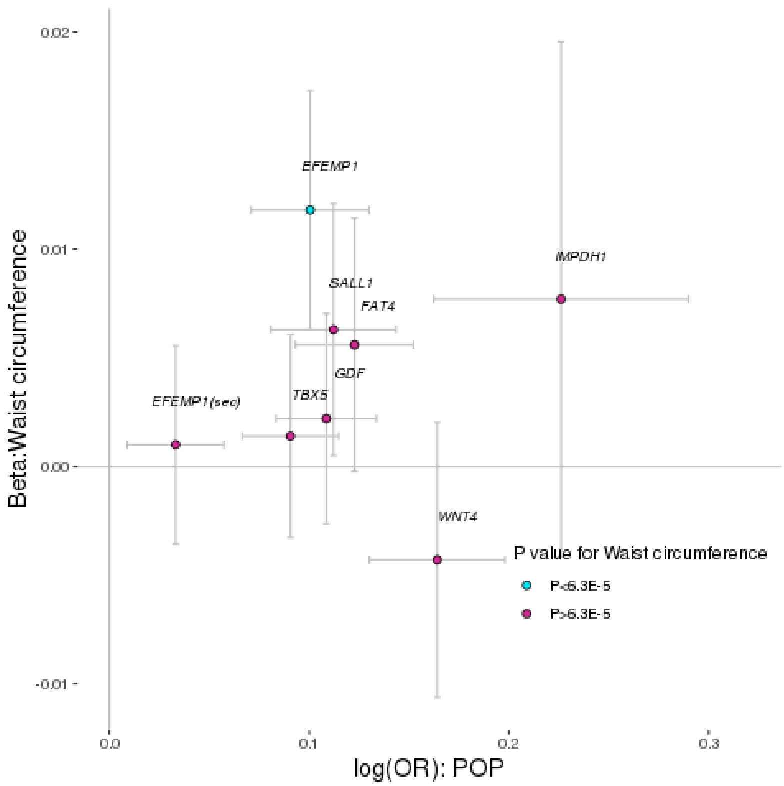

**l)** Stress incontinence

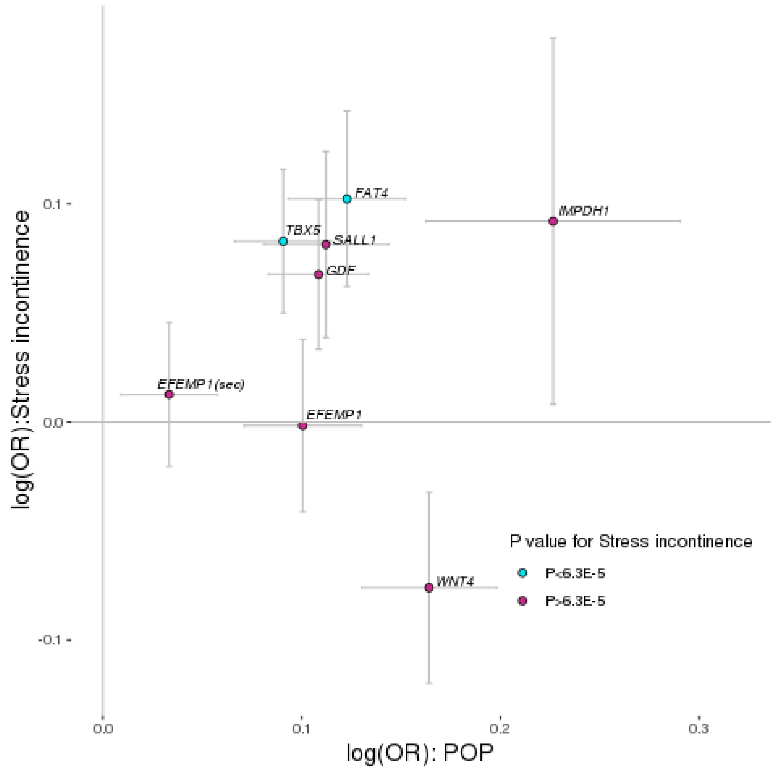


**m)** Benign prostatic hyperplasia

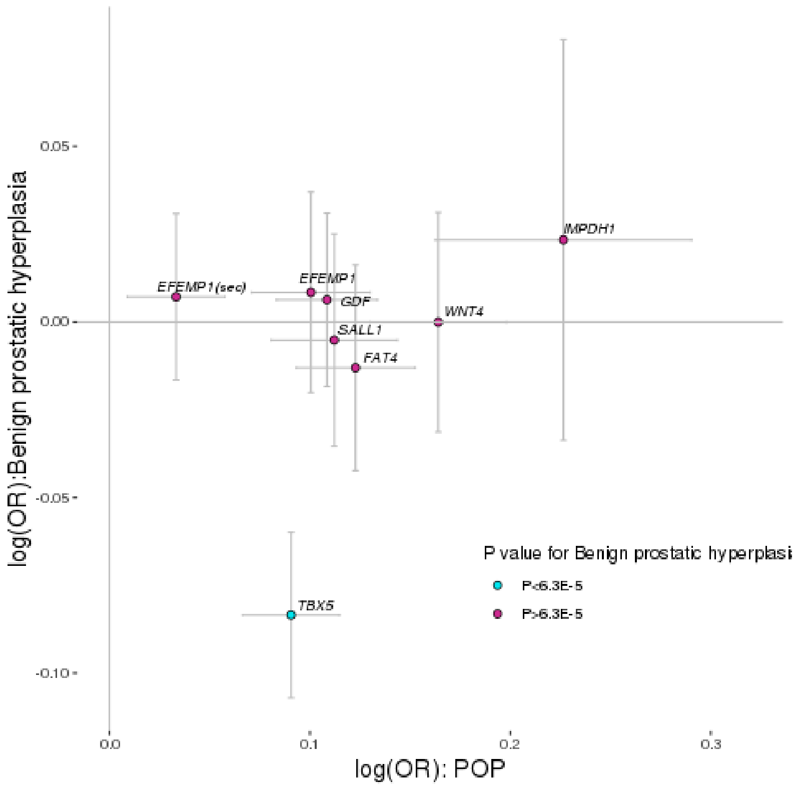

**Supplementary Figure 5 A scatter plot for the effect estimates of 152 published variants for BMI against the effect estimates of those variants on POP.** On the y-axis are the natural logarithm of the odds ratios (log(OR)) from a meta-analysis of POP in Iceland and UKB and on the x-axis are the marginal effects (betas) from the meta-analysess reported in Locke, A. E. et al., *Nature*, **518,** 197-206 (2015) and Turcot V. et al., *Nature genetics*, **50**, 766-767 (2018). Red dots denote variants not surpassing the Bonferroni-corrected threshold of a *P*-value < $3.23\times10^{-4}$ (0.05/155). Error bars denote 95% confidence intervals. Association summary results for the three reported fertility (number of children) variants also tested in this analysis specific to risk factors for POP are reported in Supplementary Data 24.

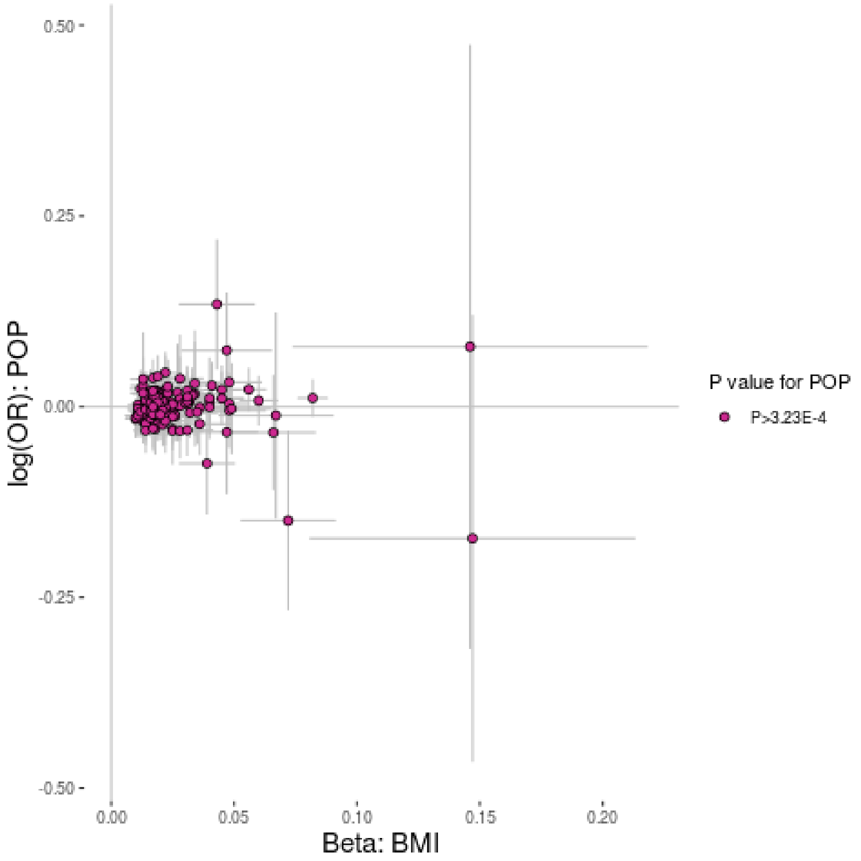

**Supplementary Figure 6 Comparison of the effect estimates of POP variants on POP against the effect estimates of those variants on two commonly reported risk factor of POP, a) BMI and b) number of children.** Effect estimates are shown for the eight index variants listed in **Table 1**. On the y-axes are betas from a meta-analysis of BMI and number of children in Iceland and UKB and on the x-axes are the log(ORs) from the meta-analysis of POP in Iceland and UKB. Red dots denote variants not surpassing the Bonferroni-corrected threshold of a *P*-value $< 6.3\times10^{-5}$ (0.05/800). Error bars denote 95% confidence intervals (Number of observations for BMI: UKB:220,310, Iceland: 51,634 ).

**a)**

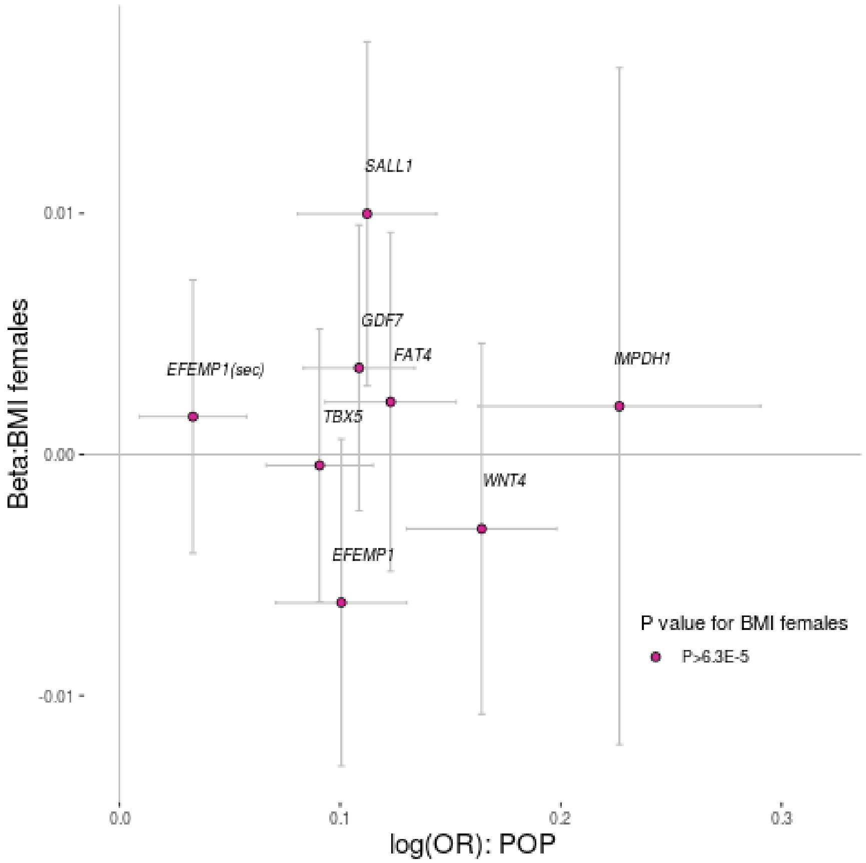

**b)**

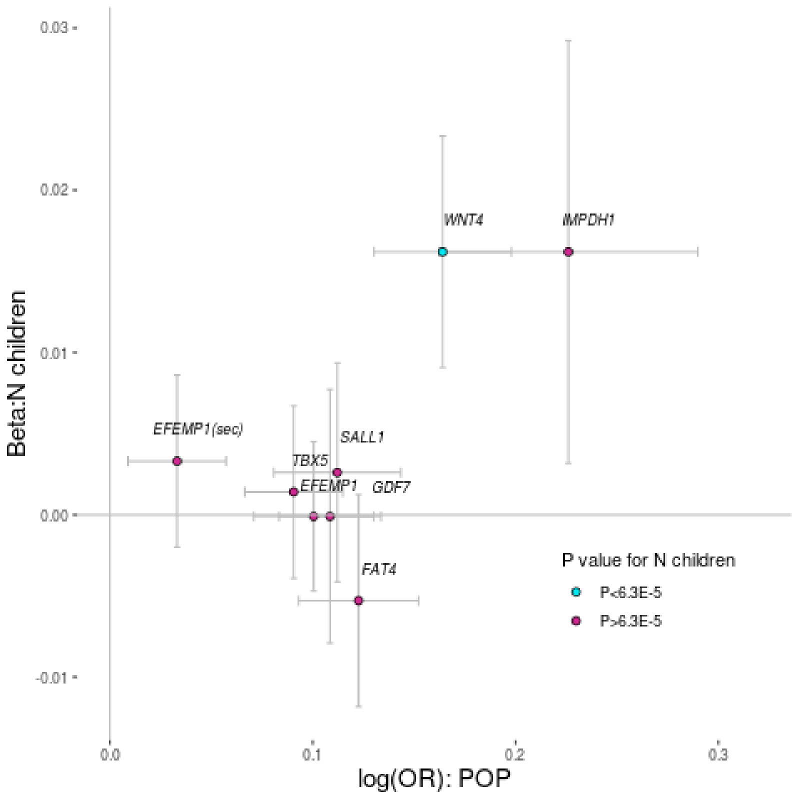

0.03
0.02
0.01
0.00
-0.01
Beta:N children
0.0
0.1
0.2
0.3
log(OR): POP
WNT4
IMPDH1
EFEMP1(sec)
SALL1
TBX5
EFEMP1
GDF7
FAT4
P value for N children
P<6.3E-5
P>6.3E-5

**Supplementary Figure 7 Regional association plots from the meta-analysis of POP around genes (+/- 250 kb windows) a) *FBLN5*, b) *HOXA11* and c) *LOXL1*, previously reported as three out of four candidate genes for POP based on mouse models**. *P*-values ($-\log_{10}$) of variant associations with POP in the meta-analysis are plotted against their NCBI Build 38 positions. As seen in panel c) rs1048661 (chr15:73927205) is the only coding variant that associates with POP at threshold $P < 1.47\times10^{-3}$ (0.05/34; sum of all coding variants tested at the three genes) ($P = 3.66\times10^{-5}$, OR=1.05) but it is explained by a stronger associated correlated (non-coding) variant at the locus; rs4886778 (chr15:73933047) (panel d)). rs12440667, a height variant associating with POP is a correlate ($r^2$=0.85) of rs4886778. The colors of the genomic variants reflect the linkage disequilibrium ($r^2$) with the annotated variants in panels c) and d) in the Icelandic data set. The grey solid line indicates recombination rates from the Icelandic recombination map for males and females (Kong, A. et al, *Nature* **467,** 1099-1103 (2010)). Known genes are shown with horizontal blue lines and exons as rectangles using data from the UCSC genes track in the UCSC Genome Browser. Arrows show the direction of transcription.

**a)**

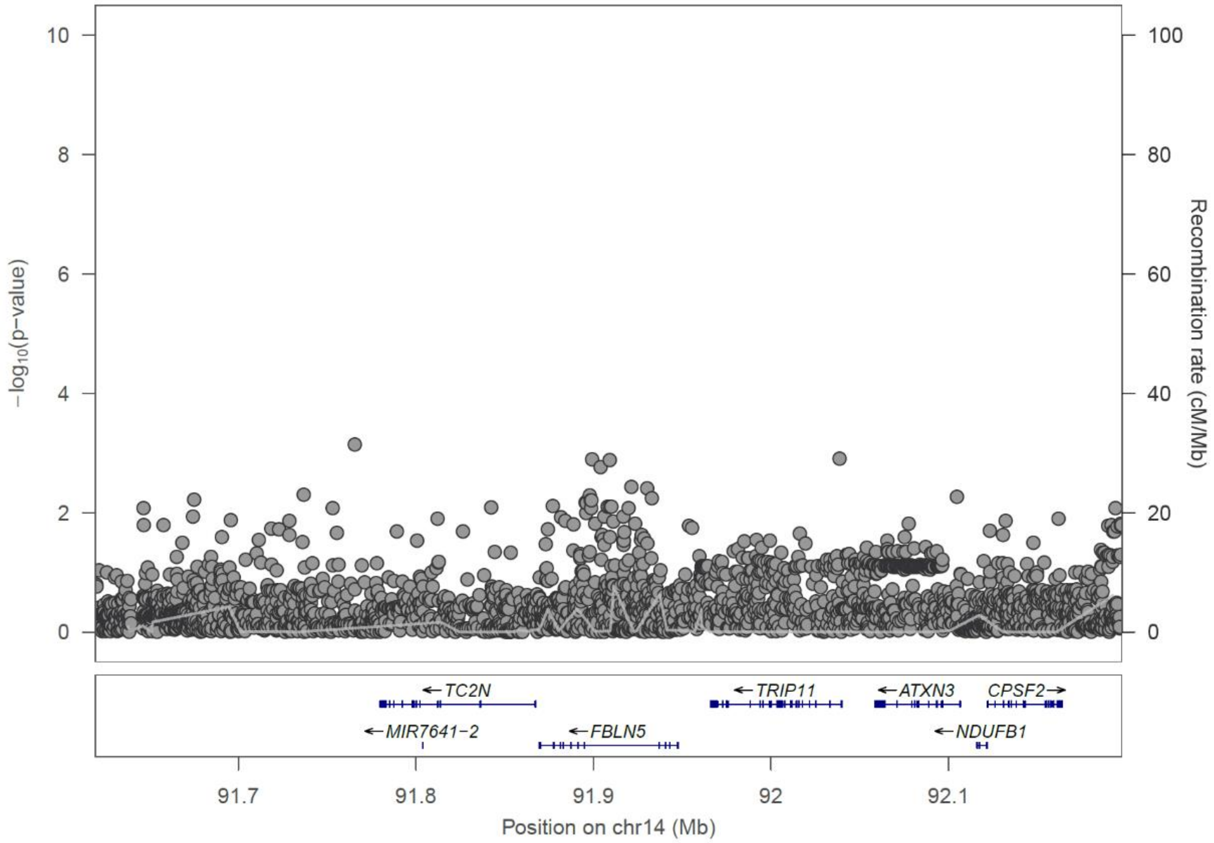

b)

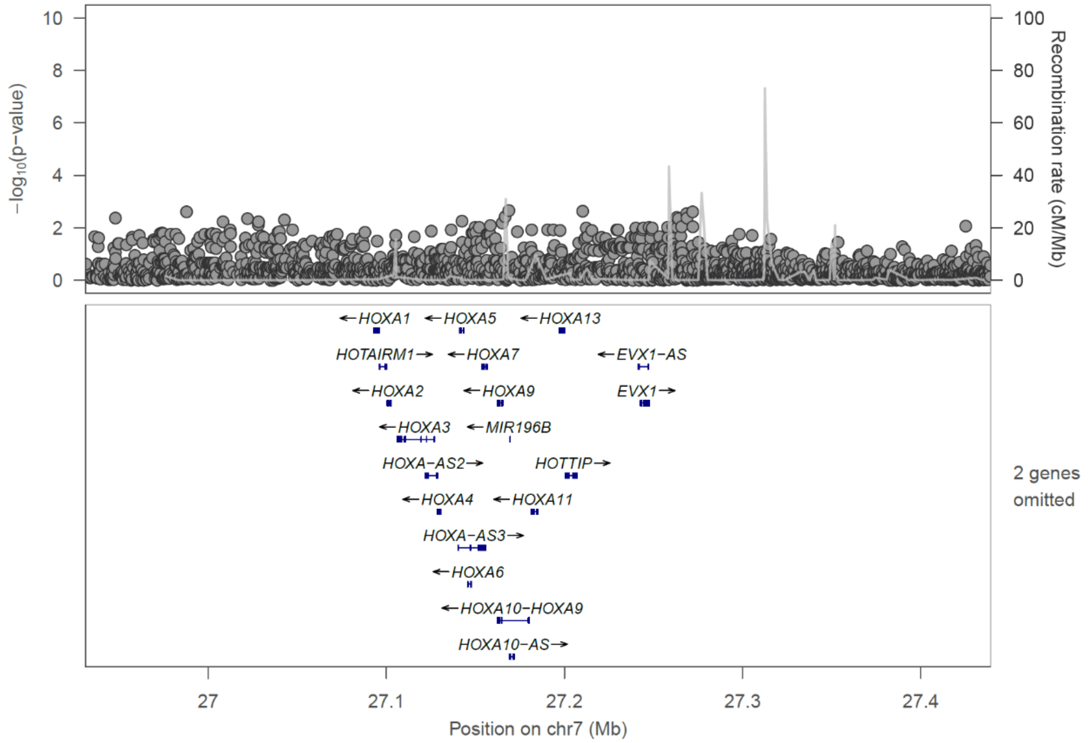

−log10(p−value)
Recombination rate (cM/Mb)
HOXA1
HOXA5
HOXA13
HOTAIRM1
HOXA7
EVX1−AS
HOXA2
HOXA9
EVX1
HOXA3
MIR196B
HOXA−AS2
HOTTIP
HOXA4
HOXA11
HOXA−AS3
HOXA6
HOXA10−HOXA9
HOXA10−AS
2 genes omitted
Position on chr7 (Mb)


c)

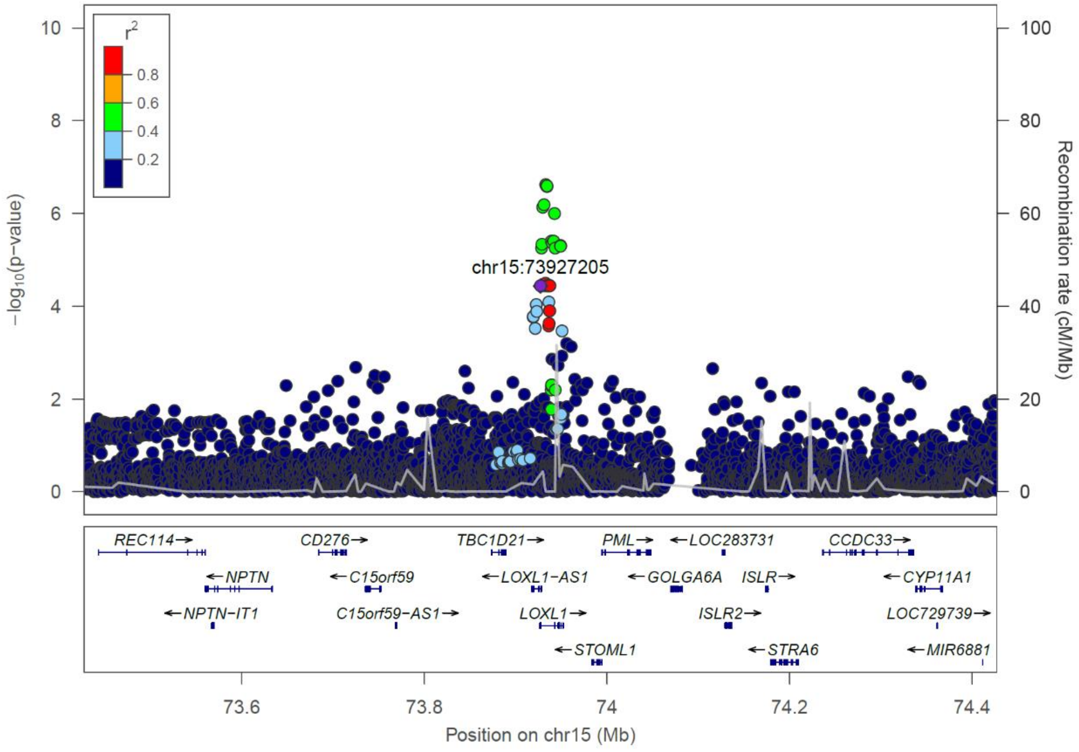

r2
0.8
0.6
0.4
0.2
−log10(p−value)
Recombination rate (cM/Mb)
chr15:73927205
REC114
CD276
TBC1D21
PML
LOC283731
CCDC33
NPTN
C15orf59
LOXL1−AS1
GOLGA6A
ISLR
CYP11A1
NPTN−IT1
C15orf59−AS1
LOXL1
ISLR2
LOC729739
STOML1
STRA6
MIR6881
Position on chr15 (Mb)

**d)**

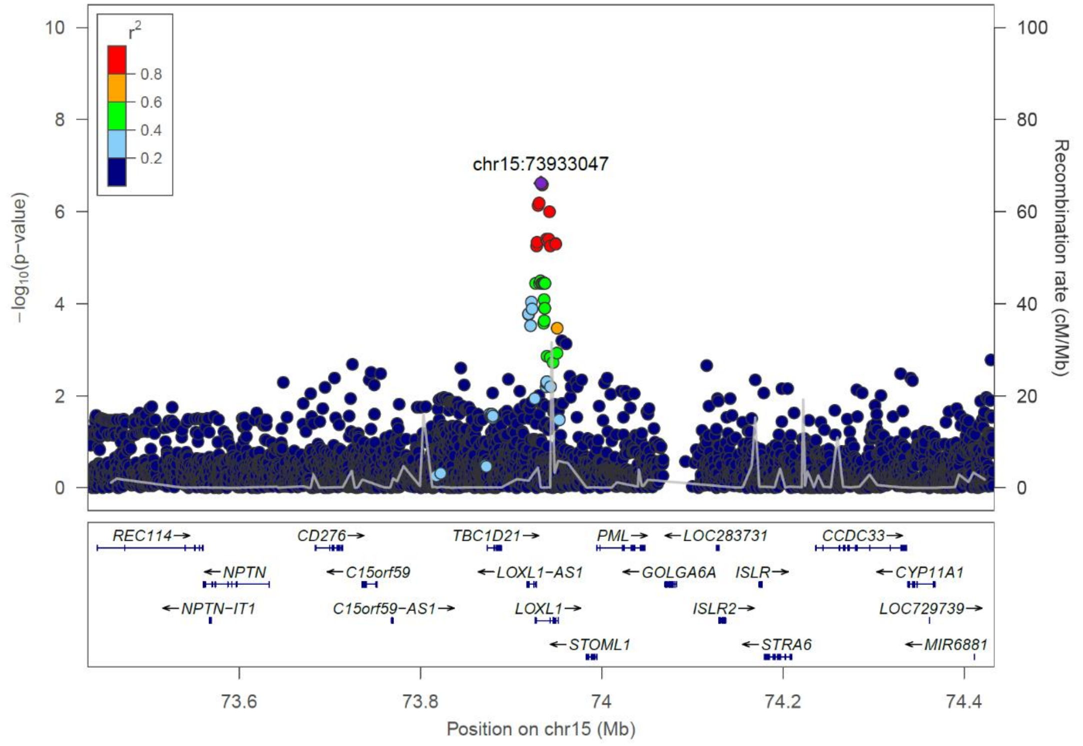

r²
0.8
0.6
0.4
0.2
chr15:73933047
−log10(p−value)
Recombination rate (cM/Mb)
0
2
4
6
8
10
0
20
40
60
80
100
REC114→
CD276→
TBC1D21→
PML→
←LOC283731
CCDC33→
←NPTN
←C15orf59
←LOXL1−AS1
←GOLGA6A
ISLR→
←CYP11A1
←NPTN−IT1
C15orf59−AS1→
LOXL1→
ISLR2→
LOC729739→
←STOML1
←STRA6
←MIR6881
73.6
73.8
74
74.2
74.4
Position on chr15 (Mb)
