# Supplementary material for: Genome-wide association identifies seven loci for pelvic organ prolapse in Iceland and the UK Biobank
Source: Commun Biol. 2020 Mar 17;3:129. doi: 10.1038/s42003-020-0857-9 (PMC7078216; doi:10.1038/s42003-020-0857-9)
Supplement: Supplementary file 4 — Reporting Summary [file 42003_2020_857_MOESM4_ESM.pdf]

nature research

Corresponding author(s): Thorhildur Olafsdottir
Kari Stefansson

Last updated by author(s): February 17th, 2020

Reporting Summary

Nature Research wishes to improve the reproducibility of the work that we publish. This form provides structure for consistency and transparency in reporting. For further information on Nature Research policies, see Authors & Referees and the Editorial Policy Checklist.

Statistics

For all statistical analyses, confirm that the following items are present in the figure legend, table legend, main text, or Methods section.

n/a	Confirmed	
☐	☒	The exact sample size (*n*) for each experimental group/condition, given as a discrete number and unit of measurement
☒	☐	A statement on whether measurements were taken from distinct samples or whether the same sample was measured repeatedly
☐	☒	The statistical test(s) used AND whether they are one- or two-sided<br>*Only common tests should be described solely by name; describe more complex techniques in the Methods section.*
☐	☒	A description of all covariates tested
☐	☒	A description of any assumptions or corrections, such as tests of normality and adjustment for multiple comparisons
☐	☒	A full description of the statistical parameters including central tendency (e.g. means) or other basic estimates (e.g. regression coefficient) AND variation (e.g. standard deviation) or associated estimates of uncertainty (e.g. confidence intervals)
☐	☒	For null hypothesis testing, the test statistic (e.g. *F*, *t*, *r*) with confidence intervals, effect sizes, degrees of freedom and *P* value noted<br>*Give P values as exact values whenever suitable.*
☒	☐	For Bayesian analysis, information on the choice of priors and Markov chain Monte Carlo settings
☒	☐	For hierarchical and complex designs, identification of the appropriate level for tests and full reporting of outcomes
☒	☐	Estimates of effect sizes (e.g. Cohen's *d*, Pearson's *r*), indicating how they were calculated

Our web collection on statistics for biologists contains articles on many of the points above.

Software and code

Policy information about availability of computer code

Data collection	*Provide a description of all commercial, open source and custom code used to collect the data in this study, specifying the version used OR state that no software was used.*
Data analysis	We used publicly available software (URLs listed below) in conjunction with the algorithms in the sequencing processing pipeline (Whole-genome sequencing, Association testing, RNA-seq mapping and analysis) as described in the Methods of our manuscript.<br>URLs:<br>BWA 0.7.10 mem, https://github.com/lh3/bwa<br>GenomeAnalysisTKLite 2.3.9, https://github.com/broadgsa/gatk/<br>Picard tools 1.117, https://broadinstitute.github.io/picard/<br>SAMtools 1.3, http://samtools.github.io/<br>Bedtools v2.25.0-76-g5e7c696z, https://github.com/arq5x/bedtools2/<br>Variant Effect Predictor https://github.com/Ensembl/ensembl-vep<br>BOLT-LMM https://data.broadinstitute.org/alkesgroup/BOLT-LMM/downloads/<br>LDSC (LD Score), https://github.com/bulik/ldsc<br><br>Variants were imputed based on the IMPUTE HMM model.<br>We used R extensively to analyze data and create plots.

For manuscripts utilizing custom algorithms or software that are central to the research but not yet described in published literature, software must be made available to editors/reviewers. We strongly encourage code deposition in a community repository (e.g. GitHub). See the Nature Research guidelines for submitting code & software for further information.

Data

Policy information about availability of data

All manuscripts must include a data availability statement. This statement should provide the following information, where applicable:

- Accession codes, unique identifiers, or web links for publicly available datasets
- A list of figures that have associated raw data
- A description of any restrictions on data availability

The Icelandic population WGS data has been deposited at the European Variant Archive under accession code PRJEB15197. The authors declare that the data supporting the findings of this study are available within the article, its Supplementary Data files and upon reasonable request. A reporting summary for this Article is available as a Supplementary Information file. The UK Biobank data can be obtained upon application (ukbiobank.ac.uk). The genome-wide association scan summary data will be made available at http://www.decode.com/summarydata

Field-specific reporting

Please select the one below that is the best fit for your research. If you are not sure, read the appropriate sections before making your selection.

[x] Life sciences [ ] Behavioural & social sciences [ ] Ecological, evolutionary & environmental sciences

For a reference copy of the document with all sections, see nature.com/documents/nr-reporting-summary-flat.pdf

Life sciences study design

All studies must disclose on these points even when the disclosure is negative.

Sample size	POP cases were identified through ICD10 and ICD9 codes from the 134,853 women from Iceland and 220,889 women from the UK with available genotypes. This yielded in total 15,010 POP cases. Details are provided in the Methods. No sample size calculation was performed as all available individuals were used for the study.
Data exclusions	No data were excluded.
Replication	We performed GWAS studies in two independent populations and combined the results. We present results for the populations independently and combined, and success of replication is reported on per-variant basis, both as individual p-values and as heterogeneity of effects between groups.
Randomization	No randomization was used and is not relevant within the context of a genome-wide association study.
Blinding	Not relevant for a genome-wide association study.

Reporting for specific materials, systems and methods

We require information from authors about some types of materials, experimental systems and methods used in many studies. Here, indicate whether each material, system or method listed is relevant to your study. If you are not sure if a list item applies to your research, read the appropriate section before selecting a response.

Materials & experimental systems

n/a	Involved in the study	
[x]	[ ]	Antibodies
[x]	[ ]	Eukaryotic cell lines
[x]	[ ]	Palaeontology
[x]	[ ]	Animals and other organisms
[ ]	[x]	Human research participants
[x]	[ ]	Clinical data

Methods

n/a	Involved in the study	
[x]	[ ]	ChIP-seq
[x]	[ ]	Flow cytometry
[x]	[ ]	MRI-based neuroimaging

Human research participants

Policy information about studies involving human research participants

Population characteristics	This is described in detail in the Methods:<br><br>"The Icelandic data originates from Landspitali – The National University Hospital Inpatient Registry from January 1983 to August 2018. A total of 3,699 women had a POP diagnosis (ICD10 code N81 or ICD9 code 618). Controls were recruited through different genetic research projects at deCODE genetics. We had genotype information for 92% of the POP cases, so that the Icelandic dataset consisted of 3,409 cases and 131,444 female controls. The UK Biobank data (UKB) consists of 11,601 cases and

209,228 controls of European ancestry (self-reported white British with similar genetic ancestry based on principal component analysis and with consistent reported and genetically determined gender), recruited between 2006 and 2010 aged 40-69, and with follow-up until 201680. The UKB cases had ICD10 code N81 in hospital inpatient records, with data dating back to between 1981 and 1997, depending on the external source (Hospital Episode Statistics from England (89% of participants), Patient Episode Database for Wales (7%) and Scottish Morbidity Record (7%)) and include primary and secondary diagnosis of POP (http://biobank.ctsu.ox.ac.uk/showcase/docs/inpatient_mapping.pdf). "

Mean age (SD) of the study population is 72(14) for cases and 57(20) for controls in the Icelandic sample and 66(7) for cases and 64(8) for controls in the UKB sample (see Supplementary Table 1 for demographic information for POP cases and controls in Iceland and the UKBiobank).

Recruitment

All cases that were identified with the procedure described above and for whom genotype information was available were used in the study. Since the risk of POP increases with age, some cases (unidentified by ICD10 N81 diagnostic code) may be misclassified as controls in the meta-analysis. This weakens the statistical power of the GWAS but should not lead to false-positive results.

Ethics oversight

The study was approved by the Icelandic National Bioethics Committee (bioethics consent number VSN 18-067) in agreement with conditions issued by the Data Protection Authority of Iceland. Written informed consent was obtained from all genotyped subjects. Personal identities relating to participants´ data and biological samples (i.e. blood samples, buccal samples, or adipose tissue samples) were encrypted by a third-party system (Identity Protection System), approved and monitored by the Data Protection Authority.

Note that full information on the approval of the study protocol must also be provided in the manuscript.
